# Screening for plant transporter function by expressing a normalized *Arabidopsis *full-length cDNA library in *Xenopus *oocytes

**DOI:** 10.1186/1746-4811-2-17

**Published:** 2006-10-27

**Authors:** Hussam H Nour-Eldin, Morten HH Nørholm, Barbara A Halkier

**Affiliations:** 1 Plant Biochemistry Laboratory, Center for Molecular Plant Physiology, The Royal Veterinary and Agricultural University, Thorvaldsensvej 40, DK-1871 Frederiksberg C, Denmark

## Abstract

**Background:**

We have developed a functional genomics approach based on expression cloning in *Xenopus *oocytes to identify plant transporter function. We utilized the full-length cDNA databases to generate a normalized library consisting of 239 full-length *Arabidopsis thaliana *transporter cDNAs. The genes were arranged into a 96-well format and optimized for expression in *Xenopus *oocytes by cloning each coding sequence into a *Xenopus *expression vector.

**Results:**

Injection of 96 *in vitro *transcribed cRNAs from the library in pools of columns and rows into oocytes and subsequent screening for glucose uptake activity identified three glucose transporters. One of these, *AtSTP13*, had not previously been experimentally characterized.

**Conclusion:**

Expression of the library in *Xenopus *oocytes, combined with uptake assays, has great potential in assignment of plant transporter function and for identifying membrane transporters for the many plant metabolites where a transporter has not yet been identified.

## Background

Higher plants produce a large number of secondary metabolites, which are transported between neighbouring cells or distant organs. For example, in tobacco plants local *de novo *synthesis accounts for less than 10% of the nicotine in aerial parts, while the remainder is transported from the roots [1]. Similarly, in *Arabidopsis thaliana *glucosinolates are transported from silique walls into seeds [2,3]. While many transporters for the major plant primary metabolites have been identified through functional complementation of yeast and bacteria, very little is known at the molecular level about transport of natural products within plants. With the completion of the *Arabidopsis *genome which presents a complete catalogue of all genes that may be expressed in the plant, it became apparent that 5–10% of the 25498 predicted proteins represent transport protein homologues [4]. Putative functions have been assigned to many of these proteins based on homology to characterized transporters in *Arabidopsis *and other organisms [5-7]. However, although phylogenetic relationships are useful for predicting structural and mechanistic properties, experimental evidence is essential for assigning function. This remains a major challenge for the majority of predicted transporter proteins in Arabidopsis.

Expression cloning in *Xenopus laevis *oocytes is a powerful tool for identifying transporters from higher eukaryotes (reviewed in [8]). The approach involves isolation of mRNA from a tissue enriched for the target transporter and expressing size-fractionated pools of this mRNA in oocytes in search of the desired uptake activity. A so-called sib selection in which positive pools are serially divided and injected into oocytes subsequently identifies the single clone. *Xenopus *oocytes are due to their low endogenous transport activity and flexible and efficient translation machinery, particularly suitable for this approach and could potentially be used to identify transporters of natural products in plants. Indeed, several successful reports exist on functional expression in *Xenopus *oocytes of plant transporters from both specific cRNAs (e.g. [9,10]) and isolated mRNA (e.g. [11,12]. However, expression cloning in *Xenopus *oocytes in its classical form has not been used successfully to isolate transporter cDNAs from higher plants. Moreover, the classical approach is highly targeted, as the isolated mRNA population is enriched for a given transporter activity through various treatments of the selected tissue. Consequently, the generated pools of cDNAs are not necessarily suitable for screening for other transport activities. We have developed a functional genomics approach to screen for function of plant transport proteins based on the classical expression cloning in *Xenopus *oocytes. We have built a library of full-length cDNAs of predicted *Arabidopsis *transporters and cloned the coding sequence (CDS) of each gene into a *Xenopus *expression vector. The library allows generation of defined and normalized cDNA pools in a reproducible manner. 96 cDNAs from the library were expressed in *Xenopus *oocytes and screened for glucose uptake activity, which identified three glucose transporters.

## Results

### Development of transporter cDNA library

The *Arabidopsis *Membrane Protein Library (AMPL) includes all predicted polytopic *Arabidopsis *genes (containing 2 or more transmembrane spanning domains) grouped into families based on sequence homology [4]. We have built a database consisting of all genes categorized as 'organic solute transporters' from the AMPL website. In addition, we selected genes from the category 'Unknown function' which had 10–14 predicted transmembrane segments (TMS), which is the most frequently occurring number of TMS in secondary transporters [13]. Out of more than 4500 genes listed in the AMPL website, our criteria identified 464 genes. At the time of search, 342 of these were categorized as organic solute transporters, and the remaining 122 were not assigned to any family. 239 of the selected AGI codes existed as full-length cDNAs deposited at either Riken Riken Bioresource Center (BRC) (226 genes) [14,15] or the Arabidopsis Gene Salk/Stanford/PGEC (SSP) consortium (13 genes) [16] [see Additional file 1]. An estimation based on available *Arabidopsis *EST sequences indicates that 320 of the 464 genes identified by our search criteria are expressed. Thus, the 239 genes available as full-length cDNAs constitute almost 75% of expressed transporters categorized as organic solute transporters or as unknowns with 10–14 TMS. According to the present annotations stated on the TAIR, TIGR and MIPS databases about 10% of the genes in the library have no known function. Based on literature searches, 15% of the genes have been assigned functions experimentally while the remaining 75% have been assigned putative functions based on sequence homology to known genes. A complete list of genes in the library and their corresponding predicted functions is included as supplementary information [see Additional file 1].

### Screening of transporter cDNA library for glucose uptake

**Figure 1:**
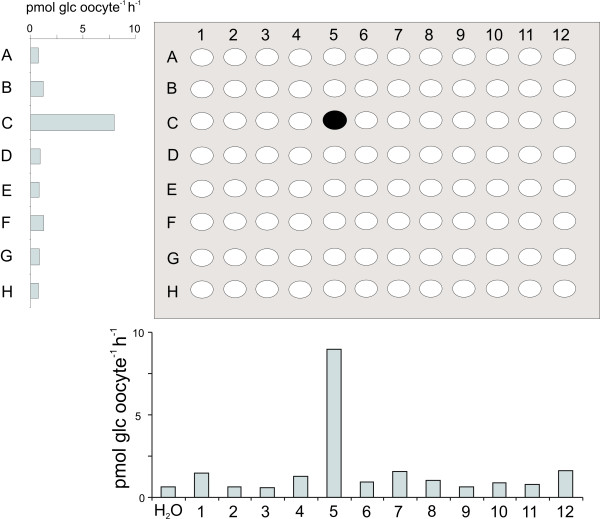
**Screening for glucose uptake activity in Xenopus oocytes expressing pools of cRNA of Arabidopsis transporter cDNAs**. cRNA of 96 genes transcribed with native plant UTRs were pooled in columns and rows and screened for glucose uptake in *Xenopus *oocytes. High glucose uptake was measured in column 5 and row C, which suggests that the marked well contains a glucose transporter.

**Figure 2:**
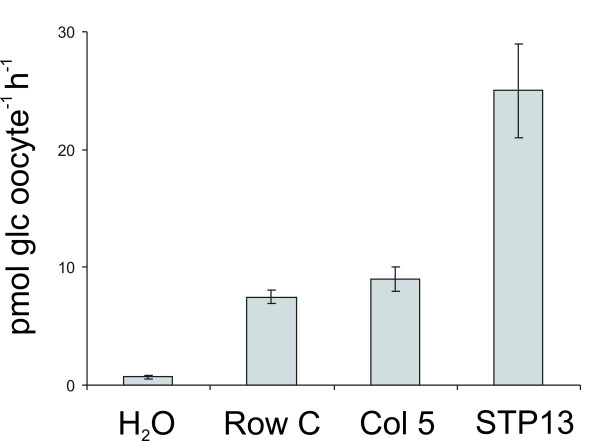
**Confirmation of glucose uptake activity by AtSTP13**. cRNA of AtSTP13 representing the single clone C5 was injected into nine oocytes. Measurement of glucose uptake by the oocytes was compared to oocytes injected with row C and column 5, respectively.

The potential of the transporter cDNA library as a tool for screening for transport activities was evaluated in a glucose and a sucrose uptake screen. We screened the first 96 cDNAs in the library among which there were several previously characterized glucose and sucrose transporters. The genes were arranged in a 96 well micro-titer plate format and combined in pools consisting of eight 'row pools' and twelve 'column pools'. Each pool was subsequently *in vitro *transcribed and injected into *Xenopus *oocytes. Glucose uptake was detected in oocytes expressing row C and column 5, which suggested that clone C5 was a glucose transporter (Fig. 1). Expression of the single clone C5 in oocytes followed by glucose uptake measurements resulted in a 4–5 times higher uptake activity compared to row C or column 5 confirming that C5 was a glucose transporter (Fig. 2). The AGI code for the C5 gene is At5g26340, which is annotated as a putative *Arabidopsis *H^+^/glucose transporter and has been named *AtSTP13 *[17]. This transporter was subsequently biochemically characterized as a high affinity hexose-specific H^+ ^symporter with an expression pattern which strongly correlates with programmed cell death [18]. The generated pools were also screened for sucrose uptake, but sucrose uptake could not be detected in any of the 20 pools (results not shown).

### The effect of different UTRs on expression in Xenopus oocytes

**Figure 3:**
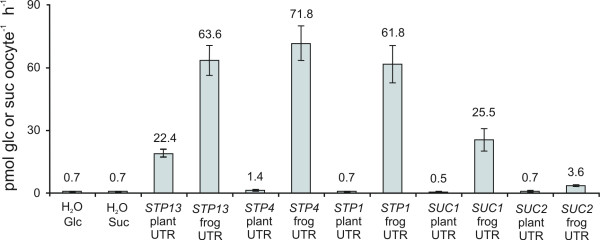
**Effect of different UTRs on gene expression in Xenopus oocytes**. CDSs of *AtSTP1, AtSTP4, AtSTP13, AtSUCI*, and *AtSUC2 *were expressed with their native plant UTRs or with *Xenopus*-specific β-globin gene UTRs. Water injected oocytes functioned as negative controls for endogenous glucose or sucrose uptake by oocytes. Assays were performed with 15 μM radio labelled glucose or sucrose (~100 μCi/μmol).

The first screen failed to identify four previously characterized glucose or sucrose transporters (*AtSTP1, AtSTP4, AtSUC1, AtSUC2*) [19-21] which were present in the 96 screened genes. The cDNAs obtained from Riken BRC contain their native untranslated regions (UTRs), which could interfere with expression in *Xenopus *oocytes and thus generate false negative results. We analyzed this possibility by cloning and expressing the CDSs of each of these four transporters as well as that of *AtSTP13 *with, respectively, their native plant UTRs and with *Xenopus-*specific UTRs. Glucose and sucrose uptake measurements on oocytes showed that the *Xenopus *UTRs resulted in a 5–30 fold increase in uptake activity for the five gene products compared to expression with the native plant UTRs (Fig. 3).

### Rescreening of transporter cDNA library optimized for Xenopus expression for glucose uptake

**Figure 4:**
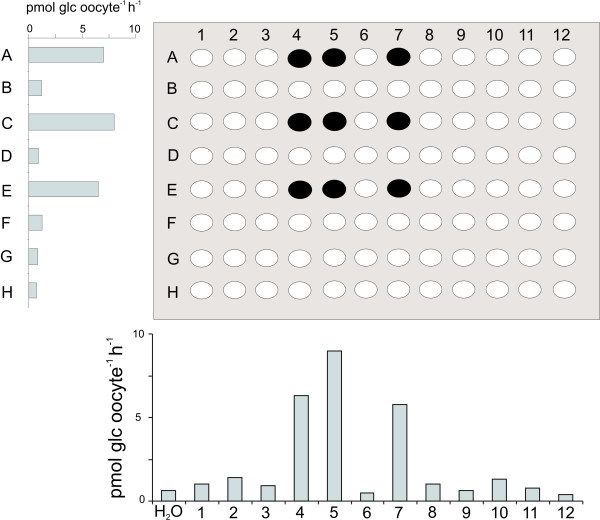
**Screening for glucose uptake in oocytes expressingpools of cRNA of Arabidopsis transporter cDNAs optimized for Xenopus expression**. cRNAs of 96 genes transcribed with *Xenopus *UTRs were pooled in columns and rows and screened for glucose uptake in oocytes. High glucose uptake was measured in row A, C and E and column 4, 5 and 7, which suggests that the marked wells, contain glucose transporters.

To overcome the interference of plant UTRs with the *Xenopus *translational machinery the library was optimized for expression in *Xenopus *oocytes by cloning the CDS of each of the 239 genes into the *Xenopus *expression vector, pNB1u, by the high-throughput USER™ cloning approach [22]. The potential of the optimized transporter cDNA library for high-throughput screening for target uptake activities was evaluated in a re-screen for glucose uptake of the same 96 cDNAs that were screened above. Glucose uptake could now be detected in oocytes expressing pools of cRNA from row A, C and E and from column 4, 5 and 7, which suggested that clones A4, A5, A7, C4, C5, C7, E4, E5 and/or E7 were glucose transporters (Fig. 4). Subsequent, expression of these clones in oocytes followed by glucose uptake measurements identified A7 (At3g19930, *AtSTP4*), C5 (At5g26340, *AtSTP13*), and E4 (At1g11260, *AtSTP1*) as encoding glucose transporters.

## Discussion

With the aim of creating a targeted functional genomics approach for assigning function to plant transporters, we have taken advantage of post-genomic tools including predictive genome annotations and full-length cDNA databases [23] to construct a normalized library of full-length *Arabidopsis *transporter cDNAs which has been optimized for expression in *Xenopus *oocytes. Storage of the cDNAs in a 96-well format enables us to pool the cDNAs in rows and columns and rapidly screen the entire library for uptake activity of a given compound. As a proof of concept, 96 cDNAs, which were known to include two previously characterized glucose transporters, *AtSTP1 *and *AtSTP4*, were screened for glucose uptake activity. While none of the positive control pools showed detectable glucose uptake, the screen identified AtSTP13 as a putative glucose transporter. As cDNAs acquired from Riken BRC contain their native plant 5' and 3' UTRs, it was expected that incompatibility between the plant UTRs and the *Xenopus *translation machinery would create false negative result. For the glucose transporters AtSTP1, AtSTP4 and AtSTP13, as well as the sucrose transporters AtSUC1 and AtSUC2, we confirmed that transcripts containing *Xenopus *β-globin gene UTRs [24] resulted in much higher levels of glucose or sucrose uptake than transcripts with native plant UTRs (Fig. 3), this has previously been shown for e.g. the *Arabidopsis *potassium channel KAT1 [25]. This indicated that although the constructed transporter library containing the native plant UTRs successfully identified AtSTP13 as a glucose transporter, it would not be optimal for an efficient screen in *Xenopus *oocytes. When we optimized the transporter library for expression in *Xenopus *oocytes by cloning each CDS into a *Xenopus *expression vector and thus replaced the native plant UTRs with *Xenopus *specific UTRs, the rate of false negative for the screen was reduced significantly as all genes annotated as glucose transporters among the screened 96 genes were now identified. Two other genes among the 96 are annotated as 'sugar transporters', but are only distantly related to the STP family which are the only characterized members of this large transporter family.

Our transporter library is expected to provide a useful tool for assignment of function to plant transporters by expression cloning in *Xenopus *oocytes. The approach has several advantages compared to previously described *Xenopus *oocyte expression cloning approaches. One strength of the approach lies in the normalized nature of the library. The individual handling and combination possibilities in columns and rows of cDNAs representing more than a hundred different *Arabidopsis *tissues ensure equal representation of weakly and strongly expressed transporters. Consequently, this permits screening for activity of weakly expressed transporters or transporters only expressed under certain conditions, without the need for isolation of RNA from enriched tissue. Furthermore, the selection of only a subgroup of genes, in the present study consisting of organic solute co-transporters and unknowns with 10–14 TMS, provides a library which is less likely to contain possible deleterious plant genes that might affect oocyte health. In addition, the exchange of native plant UTRs with *Xenopus-*specific UTRs reduces the risk of false negative results due to a possible incompatibility between the plant UTR and the *Xenopus *translational machinery. Finally, the *in vitro *transcribed pools of cRNAs can be used repeatedly in screens for transporters, provided that the desired transport activity is dependent on a single polypeptide and that the substrate is not toxic to the *Xenopus *oocytes. The type of transporters that can be identified is defined by the assays that can be performed. Simple uptake assays require a sensitive detection system to show uptake of the desired compound in to the oocytes. In this study, we performed uptake assays with a radioactively labelled glucose isotope. While this type of assay is very rapid and sensitive, it is limited by the availability of radiolabelled compounds. Other detection methods include LC-MS (liquid chromatography mass spectrometry), which is more laborious with respect to recovery of the compound from the oocytes, but poses far less limitations with regards to compound availability. Alternatively, electrophysiological measurements could be employed to detect transport of a given compound, provided the transport process is electrogenic. The screen is restricted to those transporters that fulfill the search criteria mentioned earlier as well as by the availability of full-length cDNAs. We expect to continuously expand the library with full-length transporter cDNAs as they become released, and eventually to saturate the library with all expressed secondary transporters in *Arabidopsis*. The library will be available to the scientific community through the Arabidopsis Biological Resource Center [26].

## Conclusion

We have combined post-genomic bioinformatics tools with *Xenopus *expression cloning and developed a functional genomics approach for identification of plant transporter functions. Screening of the normalized transporter library in pools is expected to enable identification of transporters for the many plant metabolites where a transporter has not yet been identified. Similar libraries can be constructed for other types of membrane proteins such as ion channels and receptors from any organism for which full-length cDNA databases exist. The approach has great potential for assignment of protein function, which is the next major challenge after the completion of genome sequences.

## Methods

### Acquisition of *Arabidopsis *full-length transporter cDNA library

Genes were selected from the *Arabidopsis *Membrane Protein Library (AMPL) database [27]. Available full-length cDNAs were obtained from databases of the Riken BRC Experimental Plant Division [28] and the *Arabidopsis *Gene Salk/Stanford/PGEC (SSP) Consortium [29]. cDNAs obtained from Riken BRC contained a full-length CDS surrounded by its specific plant UTR inserted into either the RAFL4-6 or RAFL7-11 vector [30]. cDNAs obtained from the SSP consortium contained only a CDS inserted into the Uni-vector [31]. The full-length cDNAs obtained from Riken BRC and SSP Consortium databases are derived from over hundred different *Arabidopsis thaliana *L. tissues and treatments [15].

### Subcloning of AtSTP1, AtSTP4, AtSTP13, AtSUC1 and AtSUC2 into the Xenopus expression vector, pNB1

The CDSs were PCR-amplified with CDS-specific primers from, respectively, pda05545, pda01560, pda03530, pda04389 and pda04574 plasmids from Riken.

Primer sequences:

STP1f : 5'AGCCCGGGAATGCCTGCCGGTGGATTC 3'

STP1r : 5'GAGAATTCTCAAACATGCTTCGTTCC 3'

STP4f : 5'AGCCCGGGAATGGCCGGAGGGTTCGTC 3'

STP4r : 5'GAGAATTCTCATACGGACTTCTGTTGC 3'

STP13f : 5'AGCCCGGGAATGACCGGAGGAGGATT 3'

STP13r : 5'GAAGATCTTTAAAGCCGTGTTGAAGG 3'

SUC1f : 5'AGCCCGGGAATGGGAGCCTATGAAACA 3'

SUC1r : 5'GAGAATTCCTAGTGGAATCCTCCCAT 3'

SUC2f : 5'AGCCCGGGAATGGTCAGCCATCCAATG 3'

SUC2r : 5'GAGAATTCTCAATGAAATCCCATAGT 3'

Each primer set introduced an *Xma*I and an *Eco*RI restriction site (underlined) upstream and downstream of each CDS, except for *AtSTP13 *where the reverse primer introduced a *Bgl*II site. Standard PCR was performed with *Pwo *DNA polymerase (Roche) according to the manufacturer's instructions. The PCR products were digested and ligated into a similarly digested *Xenopus *expression vector, pNB1 [32]. The constructs were confirmed by sequencing.

### Subcloning of transporter cDNA library into pNB1u

The CDSs of 239 individual cDNAs were directionally transferred into the USER™ compatible *Xenopus *expression vector pNB1u vector [22] by the improved high-throughput USER™ cloning technique [22]. pNB1u contains the 5' and 3' end UTRs of the endogenous *Xenopus *β-globin gene and has a T7 promoter situated upstream of the 5' UTR. pNB1u was prepared for cloning as described previously [22]. Briefly, 5 μg plasmid were digested with 60 U *PacI *(NEB) overnight at 37°C in a total volume of 200 μl. 20 U of *Nt.BbvCI *(NEB) and additional 20 U *Pac*I were added the next day, and the digestion was incubated for 1 h at 37°C. The linearized vector was purified with the Qiagen PCR purification kit and eluted with water. The concentration was adjusted to 10 ng/μl.

Each CDS was cloned into the pNB1u vector by PCR amplification from their respective pdaxxxxx plasmids (Riken BRC) using 0.5 U of *Pfu*Turbo^® ^Cx Hotstart DNA Polymerase (Stratagene) according to manufacturer's instructions with 30 extension-cycles.

Sequence of forward primers: 5'-ggcttaaU-gene specific sequence-3'.

Sequence of reverse primers: 5'-ggtttaaU-gene specific sequence-3'.

Tables that include the name of each gene and their corresponding Riken accession number and the sequence of all primers used to amplify the genes in the library are included in Supplementary information [see Additional file 2]. All primers were ordered from Invitrogen. PCR amplification was verified by gel electrophoresis: the PCR product was mixed with 0.75 U of USER™ reagent (NEB) and 1 μl linearized pNB1u vector at a volume ratio of 10:1 PCR product to vector. The intensity of the ethidium bromide-stained band on an agaorose gel was used to estimate the concentration of the PCR product. The mixtures were incubated for 20 min at 37°C and for 20 min at 25°C, and used to transform standard heat-shock competent *E. coli *cells. Positive colonies were identified by PCR on colonies using a vector specific primer (T7 promoter sequence) and the insert specific 3' end primer. To clone the entire library we performed two rounds of cloning. In the first round, 225 of the 239 Riken clones were successfully amplified by the ordered primers. Two colonies per plate were initially tested by PCR on colonies. For 193 clones both colonies gave the correct fragment, while 23 only gave a correct fragment in one of the tested colonies giving a total of 216 successful cloning events. Each clone was verified by sequencing and in average one out of ten clones had a PCR error which prompted picking a new colony from the plates for sequencing. In the second round of cloning we re-amplified the 23 clones which had not been successfully amplified in the first round with new primers and cloned them anew. All 23 were successfully re-amplified and were verified as described above.

### Preparation of cRNA for injection into Xenopus oocytes

Linearized DNA templates from circular plasmids for cRNA synthesis were prepared by PCR [33]. Four primer sets were used depending on the type of cDNA plasmid. One set was used for cDNAs in the Uni-vectors from the SSP consortium, two sets for cDNAs in the two RAFL vectors from Riken BRC, and one set for the CDSs in the pNB1u vector.

Primer sequences:

SSPf: 5'GTAATACGACTCACTATAGGGCCAATTAACCCTCACTAAAGGGATAACTT

SSPr : 5'TTTTTTTTTTTTTTTTTTTTTTTTTTTGAGCGCTCACAATTCTAGTCGAC

RAFL4-6f: 5'ATTAACCCTCACTAAAGGGTTGTAATACGACTCACTATAGGGGAATTGG

RAFL4-6r: 5'TTTTTTTTTTTTTTTTTTTTTTTTTGCTATGGCCCTTATGGCCGAGCTCT

RAFL7-11f: 5'ATTAACCCTCACTAAAGGGTTGTAATACGACTCACTATAGGGCAATTGG

RAFL7-11r: 5'TTTTTTTTTTTTTTTTTTTTTTTTTCCTTATGGCCGGATCCAAGAGCTCT

pNB1uf: 5'AATTAACCCTCACTAAAGGGTTGTAATACGACTCACTATAGGG

pNB1ur: 5'TTTTTTTTTTTTTTTTTTTTTTTTTTTTTATACTCAAGCTAGCCTCGAG

PCR was performed with the Phusion™ High-Fidelity DNA Polymerase (Finnzymes) according to manufacturer's instructions with 30 cycles. The PCR products were purified using MultiScreen PCRμ96 filter plates (Millipore). The concentration was normalized to 0.2 μg/μl. Each PCR product was subsequently *in vitro *transcribed as described previously [34]. Briefly, 5 μg of each PCR product was added to the following mixture: 1 × T7 transcription buffer (Fermentas), 10 mM DTT, 250 μg/ml bovine serum albumen, 1 mM rATP, 1 mM rUTP, 1 mM rCTP, 0.05 mM rGTP (Illumina), 80 U T7 RNA polymerase (Fermentas), 20 U Ribolock™ RNAse (Fermentas), 0.01 U inorganic pyrophosphatase (Fermentas), 0.06 U 3'-OMe-7 mG(5')ppp(5')G RNA cap structure analogue (NEB) in a 50 μl reaction volume. The reaction was incubated at 37°C for 30 min, after which additional rGTP was added bringing the concentration of rGTP to 1 mM. The reaction was incubated at 37°C for 3–4 h and the RNA recovered by LiCl precipitation. 100 μl ice-cold 7.5 M LiCl, 50 mM EDTA pH 8 was added and the mixture incubated at -20°C overnight, followed by centrifugation at 20,000 g for 15 min. The pellet was washed with 1 ml 70 % (v/v) ethanol, air-dried and resuspended in 10–20 μl nuclease-free water. The protocol consistently yielded approximately 40 μg RNA. The concentration of each RNA elution was adjusted to 1 μg/μl. The contents of each row and column were pooled, respectively. This resulted in 20 pools, each containing 8 or 12 cRNAs.

### Oocyte preparation and uptake assays

Oocytes were prepared as described previously [8] and injected with 50 ng cRNA. The oocytes were incubated for 2–3 d at 17–18°C before measuring transporter activity.

Uptake assays were performed in the saline buffer, Kulori (90 mM NaCl, 1 mM KCl, 1 mM CaCl_2_, 1 mM MgCl_2_, 5 mM MES, pH 5), and each assay contained nine oocytes. Reaction volume was 0.5 ml and contained 1 μCi [^14^C]glucose (303 μCi/μmol; Amersham) or [^14^C]sucrose (580 μCi/μmol; Amersham). The final concentration of glucose and sucrose was adjusted to 15 μM by addition of unlabelled glucose or sucrose. Oocytes were pre-incubated in Kulori for 5 min to ensure intracellular steady state pH [11]. Assays were stopped after 30 min by washing the oocytes four times in ice cold Kulori buffer. Oocytes were subsequently disrupted in 100 μl 10% SDS in 3 ml scintillation tubes. An aliquot (2.5 ml) EcoScint™ scintillation fluid (National Diagnostics) was added and radioactivity quantified by liquid scintillation counting.

## Competing interests

The author(s) declare that they have no competing interests.

## Authors' contributions

HHN carried out the construction of the transporter database and generation of full-length cDNA library, performed the cloning of the CDSs into the *Xenopus *oocyte expression vector and participated in the screening of the library and drafted the manuscript. MHHN participated in the design of the study, participated in the screening of the library and provided several of the constructs for testing the influence of plant UTRs on expression and helped to draft the manuscript, BAH participated in the design and coordination of the study and helped to draft the manuscript. All authors read and approved the final manuscript.

## Supplementary Material

Additional File 1 AGI code for the 239 genes in constructed full-length transporter library. This table provides a list of the genes included in the library. Predicted functions are stated. Click here for file

Additional File 2 USER cloning primers. This table provides a list of the primers used to amplify the individual CDS'es from each full length cDNA. Click here for file
