# Supplementary material for: Screening for plant transporter function by expressing a normalized Arabidopsis full-length cDNA library in Xenopus oocytes
Source: Plant Methods. 2006 Oct 27;2:17. doi: 10.1186/1746-4811-2-17 (PMC1637106; doi:10.1186/1746-4811-2-17)
Supplement: Additional File 1 — AGI code for the 239 genes in constructed full-length transporter library. This table provides a list of the genes included in the library. Predicted functions are stated. [file 1746-4811-2-17-S1.doc]

**Additional file 1**

Table 1 AGI code for the 239 genes in constructed full-length transporter library. Functions are stated as predicted on ARAMEMNON (<http://aramemnon.botanik.uni-koeln.de/>). Each AGI code is a hyperlink to its corresponding page on ARAMEMNON. If a reference exists which accounts for the functional prediction of the gene, this reference is given. If the function of the gene has been experimentally verified in the reference this is indicated by a star next to the reference number. The 96 genes which have been screened for glucose uptake in the article are the first 96 genes in the table.

**Table 1**

| Nr  Nr. | AGI code | Predicted Function | Ref |
| --- | --- | --- | --- |
| 1 | [At2g34190](http://aramemnon.botanik.uni-koeln.de/seq_view.ep?x=0&y=0&search=At2g34190+) | putative nucleobase ascorbate transporter (AtNAT2) | [1] |
| 2 | [At1g64890](http://aramemnon.botanik.uni-koeln.de/seq_view.ep?orgm=0&search=At1g64890&cat=0&term=1) | putative Folate-Biopterin Transporter | [2] |
| 3 | [AT5g65380](http://aramemnon.botanik.uni-koeln.de/seq_view.ep?orgm=0&search=AT5g65380&cat=0&term=1#bottom) | putative MATE-related efflux carrier (AtDTX27) | [3] |
| 4 | [At1g48370](http://aramemnon.botanik.uni-koeln.de/seq_view.ep?orgm=0&search=At1g48370&cat=0&term=1) | putative Fe(III)-phytosiderophore uptake mediator (AtYSL8) | [4] |
| 5 | [At1g61890](http://aramemnon.botanik.uni-koeln.de/seq_view.ep?orgm=0&search=At1g61890&cat=0&term=1) | putative MATE-related efflux carrier (AtDTX37) | [3] |
| 6 | [AT4g10770](http://aramemnon.botanik.uni-koeln.de/seq_view.ep?orgm=0&search=AT4g10770&cat=0&term=1) | oligopeptide transporter (AtOPT7) | [5]* |
| 7 | [At3g19930](http://aramemnon.botanik.uni-koeln.de/seq_view.ep?orgm=0&search=At3g19930&cat=0&term=1) | monosaccharide-proton symporter (AtSTP4) | [6] |
| 8 | [AT5g40780](http://aramemnon.botanik.uni-koeln.de/seq_view.ep?orgm=0&search=AT5g40780&cat=0&term=1) | broad-specificity high-affinity amino acid transporter (AtLHT1) | [7]* |
| 9 | [At1g08920](http://aramemnon.botanik.uni-koeln.de/seq_view.ep?orgm=0&search=At1g08920&cat=0&term=1) | sugar transporter, putative | n.a. |
| 10 | [AT5g38030](http://aramemnon.botanik.uni-koeln.de/seq_view.ep?orgm=0&search=AT5g38030&cat=0&term=1) | putative MATE-related efflux carrier (AtDTX30) | [3] |
| 11 | [AT3g53960](http://aramemnon.botanik.uni-koeln.de/seq_view.ep?orgm=0&search=AT3g53960&cat=0&term=1) | proton-dependent oligopeptide transport (POT) protein | n.a. |
| 12 | [AT3g21670](http://aramemnon.botanik.uni-koeln.de/seq_view.ep?orgm=0&search=AT3g21670&cat=0&term=1) | proton-dependent oligopeptide transport (POT) protein (ntp3) | n.a. |
| 13 | [AT5g17630](http://aramemnon.botanik.uni-koeln.de/seq_view.ep?orgm=0&search=AT5g17630&cat=0&term=1) | xylulose-5-phosphate/phosphate translocator (AtXPT) | [8]* |
| 14 | [At2g02040](http://aramemnon.botanik.uni-koeln.de/seq_view.ep?orgm=0&search=At2g02040&cat=0&term=1) | peptide transporter, histidine transporter (AtPTR2-B/AtOPT1-tNTR1 | [9]* |
| 15 | [At1g66760](http://aramemnon.botanik.uni-koeln.de/seq_view.ep?orgm=0&search=At1g66760&cat=0&term=1) | putative MATE-related efflux carrier (AtDTX9) | [3] |
| 16 | [AT3g47420](http://aramemnon.botanik.uni-koeln.de/seq_view.ep?orgm=0&search=AT3g47420&cat=0&term=1) | glycerol-3-phosphate transporter (glycerol 3-phosphate permease), putative | n.a. |
| 17 | [At1g77610](http://aramemnon.botanik.uni-koeln.de/seq_view.ep?orgm=0&search=At1g77610&cat=0&term=1) | UDP-galactose transporter, KV/A/G-group (AtUDP-GalT1) | [10] |
| 18 | [At3g54140](http://aramemnon.botanik.uni-koeln.de/seq_view.ep?orgm=0&search=At3g54140&cat=0&term=1) | peptide transporter (AtPTR1) | [11]* |
| 19 | [At3g47960](http://aramemnon.botanik.uni-koeln.de/seq_view.ep?orgm=0&search=At3g47960&cat=0&term=1) | proton-dependent oligopeptide transport (POT) protein | n.a. |
| 20 | [At1g05940](http://aramemnon.botanik.uni-koeln.de/seq_view.ep?orgm=0&search=At1g05940&cat=0&term=1) | putative cationic amino acid transporter (AtCAT9) | [12] |
| 21 | [At2g26510](http://aramemnon.botanik.uni-koeln.de/seq_view.ep?orgm=0&search=At2g26510&cat=0&term=1) | putative nucleobase ascorbate transporter | n.a. |
| 22 | [At2g21050](http://aramemnon.botanik.uni-koeln.de/seq_view.ep?orgm=0&search=At2g21050&cat=0&term=1) | putative like-AUX1 permease (AtLAX2) | [13] |
| 23 | [At5g55930](http://aramemnon.botanik.uni-koeln.de/seq_view.ep?orgm=0&search=At5g55930&cat=0&term=1) | oligopeptide transporter (AtOPT1) | [5]* |
| 24 | [At4g12030](http://aramemnon.botanik.uni-koeln.de/seq_view.ep?orgm=0&search=At4g12030&cat=0&term=1) | bile acid:sodium symporter family | n.a. |
| 25 | [At4g38250](http://aramemnon.botanik.uni-koeln.de/seq_view.ep?orgm=0&search=At4g38250&cat=0&term=1) | amino acid transporter family | n.a. |
| 26 | [At1g44750](http://aramemnon.botanik.uni-koeln.de/seq_view.ep?orgm=0&search=At1g44750&cat=0&term=1) | putative purine permease (AtPUP17) | [14] |
| 27 | [At5g27350](http://aramemnon.botanik.uni-koeln.de/seq_view.ep?orgm=0&search=At5g27350&cat=0&term=1) | putative MFS superfamily monosaccharide transporter (AtSFP1) | [15]* |
| 28 | [At1g57990](http://aramemnon.botanik.uni-koeln.de/seq_view.ep?orgm=0&search=At1g57990&cat=0&term=1) | putative purine permease (AtPUP18) | [14] |
| 29 | [At5g26340](http://aramemnon.botanik.uni-koeln.de/seq_view.ep?orgm=0&search=At5g26340&cat=0&term=1) | monosaccharide-proton symporter (AtSTP13) | [16]* |
| 30 | [At1g75220](http://aramemnon.botanik.uni-koeln.de/seq_view.ep?orgm=0&search=At1g75220&cat=0&term=1) | Unknown | n.a. |
| 31 | [At5g23810](http://aramemnon.botanik.uni-koeln.de/seq_view.ep?orgm=0&search=At5g23810&cat=0&term=1) | amino acid permease 7 (AtAAP7) | [17] |
| 32 | [At1g47670](http://aramemnon.botanik.uni-koeln.de/seq_view.ep?orgm=0&search=At1g47670&cat=0&term=1) | putative lysine/histidine transporter (AtLHT4/AtAATL1) | n.a. |
| 33 | [At4g22840](http://aramemnon.botanik.uni-koeln.de/seq_view.ep?orgm=0&search=At4g22840&cat=0&term=1) | bile acid:sodium symporter family | n.a. |
| 34 | [At1g69850](http://aramemnon.botanik.uni-koeln.de/seq_view.ep?orgm=0&search=At1g69850&cat=0&term=1) | nitrate transporter (AtNRT1.2/AtNLT1) | [18] |
| 35 | [At3g46980](http://aramemnon.botanik.uni-koeln.de/seq_view.ep?orgm=0&search=At3g46980&cat=0&term=1) | putative anion transporter (AtANTR4) | [19] |
| 36 | [At3g03720](http://aramemnon.botanik.uni-koeln.de/seq_view.ep?orgm=0&search=At3g03720&cat=0&term=1) | putative cationic amino acid transporter (AtCAT4) | [12] |
| 37 | [At1g72120](http://aramemnon.botanik.uni-koeln.de/seq_view.ep?orgm=0&search=At1g72120&cat=0&term=1) | proton-dependent oligopeptide transport (POT) protein | n.a. |
| 38 | [At1g71880](http://aramemnon.botanik.uni-koeln.de/seq_view.ep?orgm=0&search=At1g71880&cat=0&term=1) | sucrose-proton symporter/sucrose transporter 1 (AtSUC1) | [20]* |
| 39 | [At1g23080](http://aramemnon.botanik.uni-koeln.de/seq_view.ep?orgm=0&search=At1g23080&cat=0&term=1) | putative auxin efflux carrier (AtPIN7) | [21] |
| 40 | [At1g22710](http://aramemnon.botanik.uni-koeln.de/seq_view.ep?orgm=0&search=At1g22710&cat=0&term=1) | sucrose-proton symporter/sucrose transporter 2 (AtSUC2) | [22]* |
| 41 | [At3g13620](http://aramemnon.botanik.uni-koeln.de/seq_view.ep?orgm=0&search=At3g13620&cat=0&term=1) | putative neutral amino acid transport protein (AtLAT2) | n.a. |
| 42 | [At3g45680](http://aramemnon.botanik.uni-koeln.de/seq_view.ep?orgm=0&search=At3g45680&cat=0&term=1) | proton-dependent oligopeptide transport (POT) protein | n.a. |
| 43 | [At2g29650](http://aramemnon.botanik.uni-koeln.de/seq_view.ep?orgm=0&search=At2g29650&cat=0&term=1) | putative anion transporter (AtANTR1) | [19] |
| 44 | [At1g62200](http://aramemnon.botanik.uni-koeln.de/seq_view.ep?orgm=0&search=At1g62200&cat=0&term=1) | putative oligopeptide transporter | [23] |
| 45 | [At3g17650](http://aramemnon.botanik.uni-koeln.de/seq_view.ep?orgm=0&search=At3g17650&cat=0&term=1) | putative Fe(III)-phytosiderophore uptake mediator (AtYSL5) | [4] |
| 46 | [At1g47530](http://aramemnon.botanik.uni-koeln.de/seq_view.ep?orgm=0&search=At1g47530&cat=0&term=1) | putative MATE-related efflux carrier (AtDTX33) | [3] |
| 47 | [At5g16150](http://aramemnon.botanik.uni-koeln.de/seq_view.ep?orgm=0&search=At5g16150&cat=0&term=1) | putative plastidic glucose translocator (AtpGlcT) | [24]* |
| 48 | [At1g58360](http://aramemnon.botanik.uni-koeln.de/seq_view.ep?orgm=0&search=At1g58360&cat=0&term=1) | amino acid permease 1 (AtAAP1) | [25]* |
| 49 | [At1g49960](http://aramemnon.botanik.uni-koeln.de/seq_view.ep?orgm=0&search=At1g49960&cat=0&term=1) | putative nucleobase ascorbate transporter (AtNAT4) | [1] |
| 50 | [At1g77690](http://aramemnon.botanik.uni-koeln.de/seq_view.ep?orgm=0&search=At1g77690&cat=0&term=1) | putative like-AUX1 permease (AtLAX3) | [13] |
| 51 | [At4g21910](http://aramemnon.botanik.uni-koeln.de/seq_view.ep?orgm=0&search=At4g21910&cat=0&term=1) | putative MATE-related efflux carrier (AtDTX39 | [3] |
| 52 | [At1g11260](http://aramemnon.botanik.uni-koeln.de/seq_view.ep?orgm=0&search=At1g11260&cat=0&term=1) | monosaccharide-proton symporter (AtSTP1) | [26] |
| 53 | [At2g25520](http://aramemnon.botanik.uni-koeln.de/seq_view.ep?orgm=0&search=At2g25520&cat=0&term=1) | putative phosphate translocator-homolog, KD-group | n.a. |
| 54 | [At5g18840](http://aramemnon.botanik.uni-koeln.de/seq_view.ep?orgm=0&search=At5g18840&cat=0&term=1) | sugar transporter, putative | n.a. |
| 55 | [At1g69870](http://aramemnon.botanik.uni-koeln.de/seq_view.ep?orgm=0&search=At1g69870&cat=0&term=1) | proton-dependent oligopeptide transport (POT) protein | n.a. |
| 56 | [At5g46050](http://aramemnon.botanik.uni-koeln.de/seq_view.ep?orgm=0&search=At5g46050&cat=0&term=1) | stress-induced proton-dependent peptide transporter (AtPTR3) | [27] |
| 57 | [At2g40460](http://aramemnon.botanik.uni-koeln.de/seq_view.ep?orgm=0&search=At2g40460&cat=0&term=1) | proton-dependent oligopeptide transport (POT) protein | n.a. |
| 58 | [At1g17120](http://aramemnon.botanik.uni-koeln.de/seq_view.ep?orgm=0&search=At1g17120&cat=0&term=1) | putative cationic amino acid transporter (AtCAT8) | [12] |
| 59 | [At1g18880](http://aramemnon.botanik.uni-koeln.de/seq_view.ep?orgm=1&search=At1g18880&cat=0&term=1) | proton-dependent oligopeptide transport (POT) protein | n.a. |
| 60 | [At3g26590](http://aramemnon.botanik.uni-koeln.de/seq_view.ep?orgm=0&search=At3g26590&cat=0&term=1) | putative MATE-related efflux carrier (AtDTX29) | [3] |
| 61 | [At5g54800](http://aramemnon.botanik.uni-koeln.de/seq_view.ep?orgm=0&search=At5g54800&cat=0&term=1) | glucose-6-phosphate/phosphate translocator (AtGPT1) | [28] |
| 62 | [At1g80300](http://aramemnon.botanik.uni-koeln.de/seq_view.ep?orgm=0&search=At1g80300&cat=0&term=1) | plastidic ATP/ADP transporter 1 (AtAATP1/AtNTT1) | [29]* |
| 63 | [At1g15150](http://aramemnon.botanik.uni-koeln.de/seq_view.ep?orgm=0&search=At1g15150&cat=0&term=1) | putative MATE-related efflux carrier (AtDTX10) | [3] |
| 64 | [At1g09960](http://aramemnon.botanik.uni-koeln.de/seq_view.ep?orgm=0&search=At1g09960&cat=0&term=1) | low-affinity sucrose-proton symporter (AtSUT4/AtSUC4) | [30]* |
| 65 | [At3g30390](http://aramemnon.botanik.uni-koeln.de/seq_view.ep?orgm=0&search=At3g30390&cat=0&term=1) | amino acid transporter family | n.a. |
| 66 | [At1g80510](http://aramemnon.botanik.uni-koeln.de/seq_view.ep?orgm=0&search=At1g80510&cat=0&term=1) | amino acid transporter family | n.a. |
| 67 | [At1g68100](http://aramemnon.botanik.uni-koeln.de/seq_view.ep?orgm=0&search=At1g68100&cat=0&term=1) | protein required for auxin conjugate sensitivity (AtIAR 1) | [31]* |
| 68 | [At1g16390](http://aramemnon.botanik.uni-koeln.de/seq_view.ep?orgm=0&search=At1g16390&cat=0&term=1) | organic cation transporter-related | n.a. |
| 69 | [At3g48200](http://aramemnon.botanik.uni-koeln.de/seq_view.ep?orgm=0&search=At3g48200&cat=0&term=1) | Unknown | n.a. |
| 70 | [At1g32080](http://aramemnon.botanik.uni-koeln.de/seq_view.ep?orgm=0&search=At1g32080&cat=0&term=1) | Unknown | n.a. |
| 71 | [At5g27730](http://aramemnon.botanik.uni-koeln.de/seq_view.ep?orgm=0&search=At5g27730&cat=0&term=1) | Unknown | n.a. |
| 72 | [At5g64500](http://aramemnon.botanik.uni-koeln.de/seq_view.ep?orgm=0&search=At5g64500&cat=0&term=1) | Unknown | n.a. |
| 73 | [At5g54860](http://aramemnon.botanik.uni-koeln.de/seq_view.ep?orgm=0&search=At5g64500&cat=0&term=1) | Unknown | n.a. |
| 74 | [At1g70330](http://aramemnon.botanik.uni-koeln.de/seq_view.ep?orgm=0&search=At1g70330&cat=0&term=1) | proton-dependent concentrative adenosine transporter (AtENT1) | [32]* |
| 75 | [At1g71090](http://aramemnon.botanik.uni-koeln.de/seq_view.ep?orgm=0&search=At1g71090&cat=0&term=1) | Putative auxin efflux carrier protein | n.a. |
| 76 | [At4g35870](http://aramemnon.botanik.uni-koeln.de/seq_view.ep?orgm=0&search=At4g35870&cat=0&term=1) | Unknown | n.a. |
| 77 | [At5g33320](http://aramemnon.botanik.uni-koeln.de/seq_view.ep?orgm=0&search=At5g33320&cat=0&term=1) | phosphoenolpyruvate/phosphate translocator (AtPPT1) | [33]* |
| 78 | [At1g63690](http://aramemnon.botanik.uni-koeln.de/seq_view.ep?x=0&y=0&search=At1g63690) | protease-associated protein | n.a. |
| 79 | [At5g13750](http://aramemnon.botanik.uni-koeln.de/seq_view.ep?orgm=0&search=At5g13750&cat=0&term=1) | Unknown | n.a. |
| 80 | [At4g00350](http://aramemnon.botanik.uni-koeln.de/seq_view.ep?orgm=0&search=At4g00350&cat=0&term=1) | putative MATE-related efflux carrier (AtDTX34) | [3] |
| 81 | [At5g13400](http://aramemnon.botanik.uni-koeln.de/seq_view.ep?orgm=0&search=At5g13400&cat=0&term=1) | proton-dependent oligopeptide transport (POT) protein | n.a. |
| 82 | [At5g09220](http://aramemnon.botanik.uni-koeln.de/seq_view.ep?orgm=0&search=At5g09220&cat=0&term=1) | amino acid permease 2 (AtAAP2) | [34] |
| 83 | [At1g57990](http://aramemnon.botanik.uni-koeln.de/seq_view.ep?orgm=0&search=At1g57990&cat=0&term=1) | putative purine permease (AtPUP18) | n.a. |
| 84 | [At1g12480](http://aramemnon.botanik.uni-koeln.de/seq_view.ep?orgm=0&search=At1g12480&cat=0&term=1) | C4-dicarboxylate transporter/malic acid transport protein | n.a. |
| 85 | [At2g03520](http://aramemnon.botanik.uni-koeln.de/seq_view.ep?orgm=0&search=At2g03520&cat=0&term=1) | ureide permease (AtUPS4) | [35] |
| 86 | [At4g22840](http://aramemnon.botanik.uni-koeln.de/seq_view.ep?orgm=0&search=At4g22840&cat=0&term=1) | bile acid:sodium symporter family | n.a. |
| 87 | [At1g12640](http://aramemnon.botanik.uni-koeln.de/seq_view.ep?orgm=0&search=At1g12640&cat=0&term=1) | membrane bound O-acyl transferase (MBOAT) family | n.a. |
| 88 | [At1g33080](http://aramemnon.botanik.uni-koeln.de/seq_view.ep?orgm=0&search=At1g33080&cat=0&term=1) | putative MATE-related efflux carrier (AtDTX23) | [3] |
| 89 | [At4g13345](http://aramemnon.botanik.uni-koeln.de/seq_view.ep?orgm=0&search=At4g13345&cat=0&term=1) | TMS membrane protein / tumour differentially expressed (TDE) family | n.a. |
| 90 | [At5g64290](http://aramemnon.botanik.uni-koeln.de/seq_view.ep?orgm=0&search=At5g64290&cat=0&term=1) | plastidic glutamate/malate-translocator (AtDiT2.1/AtpDCT1) | [36]* |
| 91 | [At3g25410](http://aramemnon.botanik.uni-koeln.de/seq_view.ep?orgm=0&search=At3g25410&cat=0&term=1) | bile acid:sodium symporter family | n.a. |
| 92 | [At3g25410](http://aramemnon.botanik.uni-koeln.de/seq_view.ep?orgm=0&search=At3g25410&cat=0&term=1) | bile acid:sodium symporter family | n.a. |
| 93 | [At5g14850](http://aramemnon.botanik.uni-koeln.de/seq_view.ep?orgm=0&search=At5g14850&cat=0&term=1) | dolichyl-phosphate-mannose--glycolipid alpha-mannosyltransferase-like protein | n.a. |
| 94 | [At1g63010](http://aramemnon.botanik.uni-koeln.de/seq_view.ep?orgm=0&search=At1g63010&cat=0&term=1) | SPX (SYG1/Pho81/XPR1) domain protein | n.a. |
| 95 | [At1g79820](http://aramemnon.botanik.uni-koeln.de/seq_view.ep?orgm=0&search=At1g79820&cat=0&term=1) | Golgi-localized hexose transporter (AtSGB1) | [37]* |
| 96 | [At5g55960](http://aramemnon.botanik.uni-koeln.de/seq_view.ep?orgm=0&search=At5g55960&cat=0&term=1) | Unknown | n.a. |
| 97 | [At5g26820](http://aramemnon.botanik.uni-koeln.de/seq_view.ep?orgm=0&search=At5g26820&cat=0&term=1) | putative iron-regulated transporter (AtIREG3) | [38] |
| 98 | [At2g21340](http://aramemnon.botanik.uni-koeln.de/seq_view.ep?orgm=0&search=At2g21340&cat=0&term=1) | putative MATE-related efflux carrier (AtDTX46) | [3] |
| 99 | [At5g13760](http://aramemnon.botanik.uni-koeln.de/seq_view.ep?orgm=0&search=At5g13760&cat=0&term=1) | Unknown | n.a. |
| 100 | [At5g13740](http://aramemnon.botanik.uni-koeln.de/seq_view.ep?orgm=0&search=At5g13740&cat=0&term=1) | Unknown | n.a. |
| 101 | [At4g27720](http://aramemnon.botanik.uni-koeln.de/seq_view.ep?orgm=0&search=At4g27720&cat=0&term=1) | Unknown | n.a. |
| 102 | [At1g72130](http://aramemnon.botanik.uni-koeln.de/seq_view.ep?orgm=0&search=At1g72130&cat=0&term=1) | proton-dependent oligopeptide transport (POT) protein | n.a. |
| 103 | [At5g03555](http://aramemnon.botanik.uni-koeln.de/seq_view.ep?orgm=0&search=At5g03555&cat=0&term=1) | permease, cytosine/purines, uracil, thiamine, allantoin protein | n.a. |
| 104 | [At3g56200](http://aramemnon.botanik.uni-koeln.de/seq_view.ep?orgm=0&search=At3g56200&cat=0&term=1) | amino acid transporter family | n.a. |
| 105 | [At4g27970](http://aramemnon.botanik.uni-koeln.de/seq_view.ep?orgm=0&search=At4g27970&cat=0&term=1) | C4-dicarboxylate transporter/malic acid transport protein | n.a. |
| 106 | [At5g38460](http://aramemnon.botanik.uni-koeln.de/seq_view.ep?orgm=0&search=At5g38460&cat=0&term=1) | ALG6, ALG8 glycosyltransferase family | n.a. |
| 107 | [At5g10190](http://aramemnon.botanik.uni-koeln.de/seq_view.ep?orgm=0&search=At5g10190&cat=0&term=1) | Unknown | n.a. |
| 108 | [At2g36630](http://aramemnon.botanik.uni-koeln.de/seq_view.ep?orgm=0&search=At2g36630&cat=0&term=1) | Unknown | n.a. |
| 109 | [At4g36790](http://aramemnon.botanik.uni-koeln.de/seq_view.ep?orgm=0&search=At4g36790&cat=0&term=1) | Unknown | n.a. |
| 110 | [At2g01170](http://aramemnon.botanik.uni-koeln.de/seq_view.ep?orgm=0&search=At2g01170&cat=0&term=1) | amino acid permease family | n.a. |
| 111 | [At5g46340](http://aramemnon.botanik.uni-koeln.de/seq_view.ep?orgm=0&search=At5g46340&cat=0&term=1) | O-acetyltransferase-related | n.a. |
| 112 | [At5g49990](http://aramemnon.botanik.uni-koeln.de/seq_view.ep?orgm=0&search=At5g49990&cat=0&term=1) | putative nucleobase ascorbate transporter (AtNAT5) | [1] |
| 113 | [At3g10960](http://aramemnon.botanik.uni-koeln.de/seq_view.ep?orgm=0&search=At3g10960&cat=0&term=1) | Unknown | n.a. |
| 114 | [At2g04100](http://aramemnon.botanik.uni-koeln.de/seq_view.ep?orgm=0&search=At2g04100&cat=0&term=1) | putative MATE-related efflux carrier (AtDTX6) | [3] |
| 115 | [At3g59310](http://aramemnon.botanik.uni-koeln.de/seq_view.ep?orgm=0&search=At3g59310&cat=0&term=1) | Unknown | n.a. |
| 116 | [At3g60070](http://aramemnon.botanik.uni-koeln.de/seq_view.ep?orgm=0&search=At3g60070&cat=0&term=1) | lactose permease-related | n.a. |
| 117 | [At4g22990](http://aramemnon.botanik.uni-koeln.de/seq_view.ep?orgm=0&search=At4g22990&cat=0&term=1) | SPX (SYG1/Pho81/XPR1) domain protein | n.a. |
| 118 | [At1g68570](http://aramemnon.botanik.uni-koeln.de/seq_view.ep?orgm=0&search=At1g68570&cat=0&term=1) | proton-dependent oligopeptide transport (POT) protein | n.a. |
| 119 | [At5g52540](http://aramemnon.botanik.uni-koeln.de/seq_view.ep?orgm=0&search=At5g52540&cat=0&term=1) | Unknown | n.a. |
| 120 | [At5g51710](http://aramemnon.botanik.uni-koeln.de/seq_view.ep?orgm=0&search=At5g51710&cat=0&term=1) | putative potassium efflux antiporter, CPA2 subfamily (AtKEA5) | [39] |
| 121 | [At2g01420](http://aramemnon.botanik.uni-koeln.de/seq_view.ep?orgm=0&search=At2g01420&cat=0&term=1) | auxin transporter splice variant b (AtPIN4) | [40]* |
| 122 | [At5g63850](http://aramemnon.botanik.uni-koeln.de/seq_view.ep?orgm=0&search=At5g63850&cat=0&term=1) | amino acid permease 4 (AtAAP4) | [41]* |
| 123 | [At4g04340](http://aramemnon.botanik.uni-koeln.de/seq_view.ep?orgm=0&search=At4g04340&cat=0&term=1) | ERD4 protein-related | n.a. |
| 124 | [At5g02410](http://aramemnon.botanik.uni-koeln.de/seq_view.ep?orgm=0&search=At5g02410&cat=0&term=1) | DIE2/ALG10 family | n.a. |
| 125 | [At2g27810](http://aramemnon.botanik.uni-koeln.de/seq_view.ep?orgm=0&search=At2g27810&cat=0&term=1) | putative nucleobase ascorbate transporter (AtNAT12) | [1] |
| 126 | [At4g35300](http://aramemnon.botanik.uni-koeln.de/seq_view.ep?orgm=0&search=At4g35300&cat=0&term=1) | putative monosaccharide transporter, large central loop (AtAZT3/AttMT2) | n.a. |
| 127 | [At1g18010](http://aramemnon.botanik.uni-koeln.de/seq_view.ep?orgm=0&search=At1g18010&cat=0&term=1) | Unknown | n.a. |
| 128 | [At1g50430](http://aramemnon.botanik.uni-koeln.de/seq_view.ep?orgm=0&search=At1g50430&cat=0&term=1) | sterol delta-7 reductase, 7-dehydrocholesterol reductase (AtDWF5) | n.a. |
| 129 | [At3g16180](http://aramemnon.botanik.uni-koeln.de/seq_view.ep?orgm=0&search=At3g16180&cat=0&term=1) | proton-dependent oligopeptide transport (POT) protein | n.a. |
| 130 | [At1g15500](http://aramemnon.botanik.uni-koeln.de/seq_view.ep?orgm=0&search=At1g15500&cat=0&term=1) | plastidic ATP/ADP transporter 2 (AtAATP2/AtNTT2) | [29]* |
| 131 | [At5g17700](http://aramemnon.botanik.uni-koeln.de/seq_view.ep?orgm=0&search=At5g17700&cat=0&term=1) | putative MATE-related efflux carrier (AtDTX25) | [3] |
| 132 | [At3g02690](http://aramemnon.botanik.uni-koeln.de/seq_view.ep?orgm=0&search=At3g02690&cat=0&term=1) | integral membrane protein | (9) |
| 133 | [At5g57090](http://aramemnon.botanik.uni-koeln.de/seq_view.ep?orgm=0&search=At5g57090&cat=0&term=1) | auxin transporter/auxin efflux carrier (AtEIR1/AtAGR1/AtPIN2) | [42] |
| 134 | [At1g30220](http://aramemnon.botanik.uni-koeln.de/seq_view.ep?orgm=0&search=At1g30220&cat=0&term=1) | putative inositol/polyol (cyclic)-proton symporter (AtINT2) | [43] |
| 135 | [At3g21690](http://aramemnon.botanik.uni-koeln.de/seq_view.ep?orgm=0&search=At3g21690&cat=0&term=1) | putative MATE-related efflux carrier (AtDTX40) | [3] |
| 136 | [At1g76520](http://aramemnon.botanik.uni-koeln.de/seq_view.ep?orgm=0&search=At1g76520&cat=0&term=1) | auxin efflux carrier protein | n.a. |
| 137 | [At1g77380](http://aramemnon.botanik.uni-koeln.de/seq_view.ep?orgm=0&search=At1g77380&cat=0&term=1) | amino acid permease 3 (AtAAP3) | [44] |
| 138 | [At2g26690](http://aramemnon.botanik.uni-koeln.de/seq_view.ep?orgm=0&search=At2g26690&cat=0&term=1) | low-affinity nitrate transporter (AtNRT1.4) | [45] |
| 139 | [At5g65000](http://aramemnon.botanik.uni-koeln.de/seq_view.ep?orgm=0&search=At5g65000&cat=0&term=1) | nucleotide-sugar transporter family | n.a. |
| 140 | [At1g57600](http://aramemnon.botanik.uni-koeln.de/seq_view.ep?orgm=0&search=At1g57600&cat=0&term=1) | membrane bound O-acyl transferase (MBOAT) family | n.a. |
| 141 | [At1g55620](http://aramemnon.botanik.uni-koeln.de/seq_view.ep?orgm=0&search=At1g55620&cat=0&term=1) | voltage-gated chloride channel protein (AtCLC-f) | n.a. |
| 142 | [At1g63050](http://aramemnon.botanik.uni-koeln.de/seq_view.ep?orgm=0&search=At1g63050&cat=0&term=1) | membrane bound O-acyl transferase (MBOAT) family | n.a. |
| 143 | [At5g64410](http://aramemnon.botanik.uni-koeln.de/seq_view.ep?orgm=0&search=At5g64410&cat=0&term=1) | oligopeptide transporter (AtOPT4) | [46]* |
| 144 | [At3g21620](http://aramemnon.botanik.uni-koeln.de/seq_view.ep?orgm=0&search=At3g21620&cat=0&term=1) | ERD4 protein-related | n.a. |
| 145 | [At5g04770](http://aramemnon.botanik.uni-koeln.de/seq_view.ep?orgm=0&search=At5g04770&cat=0&term=1) | putative cationic amino acid transporter (AtCAT6) | + |
| 146 | [At3g59360](http://aramemnon.botanik.uni-koeln.de/seq_view.ep?orgm=0&search=At3g59360&cat=0&term=1) | putative UDP-galactose/UDP-glucose transporter (AtUTr6) | [47] |
| 147 | [At1g59740](http://aramemnon.botanik.uni-koeln.de/seq_view.ep?orgm=0&search=At1g59740&cat=0&term=1) | proton-dependent oligopeptide transport (POT) protein | n.a. |
| 148 | [At4g26590](http://aramemnon.botanik.uni-koeln.de/seq_view.ep?orgm=0&search=At4g26590&cat=0&term=1) | oligopeptide transporter (AtOPT5) | [5]* |
| 149 | [At3g55640](http://aramemnon.botanik.uni-koeln.de/seq_view.ep?orgm=0&search=At3g55640&cat=0&term=1) | putative mitochondrial Ca2+ dependent carrier | n.a. |
| 150 | [At1g15180](http://aramemnon.botanik.uni-koeln.de/seq_view.ep?orgm=0&search=At1g15180&cat=0&term=1) | putative MATE-related efflux carrier (AtDTX13) | [3] |
| 151 | [At5g52450](http://aramemnon.botanik.uni-koeln.de/seq_view.ep?orgm=0&search=At5g52450&cat=0&term=1) | putative MATE-related efflux carrier (AtDTX16) | [3] |
| 152 | [At5g46110](http://aramemnon.botanik.uni-koeln.de/seq_view.ep?orgm=0&search=At5g46110&cat=0&term=1) | phosphate/triose-phosphate translocator (AtTPT) | [48] |
| 153 | [At1g10540](http://aramemnon.botanik.uni-koeln.de/seq_view.ep?orgm=0&search=At1g10540&cat=0&term=1) | putative nucleobase ascorbate transporter (AtNAT8) | [1] |
| 154 | [At1g27040](http://aramemnon.botanik.uni-koeln.de/seq_view.ep?orgm=0&search=At1g27040&cat=0&term=1) | putative nitrate transporter | n.a. |
| 155 | [At3g05400](http://aramemnon.botanik.uni-koeln.de/seq_view.ep?orgm=0&search=At3g05400&cat=0&term=1) | sugar transporter, putative | n.a. |
| 156 | [At4g32400](http://aramemnon.botanik.uni-koeln.de/seq_view.ep?orgm=0&search=At4g32400&cat=0&term=1) | putative mitochondrial adenylate translocator | n.a. |
| 157 | [At1g73590](http://aramemnon.botanik.uni-koeln.de/seq_view.ep?orgm=0&search=At1g73590&cat=0&term=1) | putative auxin efflux carrier PIN-FORMED 1 (AtPIN1) | [49] |
| 158 | [At5g62890](http://aramemnon.botanik.uni-koeln.de/seq_view.ep?orgm=0&search=At1g73590&cat=0&term=1) | putative nucleobase ascorbate transporter (AtNAT6) | [1] |
| 159 | [At1g78560](http://aramemnon.botanik.uni-koeln.de/seq_view.ep?orgm=0&search=At1g78560&cat=0&term=1) | bile acid:sodium symporter family | n.a. |
| 160 | [At1g44100](http://aramemnon.botanik.uni-koeln.de/seq_view.ep?orgm=0&search=At1g44100&cat=0&term=1) | amino acid permease 5 (AtAAP5) | [50]* |
| 161 | [At4g24120](http://aramemnon.botanik.uni-koeln.de/seq_view.ep?orgm=0&search=At4g24120&cat=0&term=1) | putative Fe(III)-phytosiderophore uptake mediator (AtYSL1) | [4]* |
| 162 | [At1g12110](http://aramemnon.botanik.uni-koeln.de/seq_view.ep?orgm=0&search=At1g12110&cat=0&term=1) | dual-affinity nitrate transporter (AtNRT1.1/AtCHL1) | [51]* |
| 163 | [At3g23560](http://aramemnon.botanik.uni-koeln.de/seq_view.ep?orgm=0&search=At3g23560&cat=0&term=1) | MATE-related multidrug efflux transporter (AtALF5/AtDTX19) | [3] |
| 164 | [At2g48020](http://aramemnon.botanik.uni-koeln.de/seq_view.ep?orgm=0&search=At2g48020&cat=0&term=1) | sugar transporter, putative | n.a. |
| 165 | [At3g27020](http://aramemnon.botanik.uni-koeln.de/seq_view.ep?orgm=0&search=At3g27020&cat=0&term=1) | putative Fe(III)-phytosiderophore uptake mediator (AtYSL6) | [4] |
| 166 | [At1g48640](http://aramemnon.botanik.uni-koeln.de/seq_view.ep?orgm=0&search=At1g48640&cat=0&term=1) | lysine and histidine specific transporter, putative | n.a. |
| 167 | [At5g01500](http://aramemnon.botanik.uni-koeln.de/seq_view.ep?orgm=0&search=At5g01500&cat=0&term=1) | putative mitochondrial carrier | n.a. |
| 168 | [At3g18830](http://aramemnon.botanik.uni-koeln.de/seq_view.ep?orgm=0&search=At3g18830&cat=0&term=1) | broad-spectrum H(+)-symporter for linear polyols (AtPLT5) | [52] |
| 169 | [At2g41700](http://aramemnon.botanik.uni-koeln.de/seq_view.ep?orgm=0&search=At2g41700&cat=0&term=1) | putative ABC1 homolog (AtAOH1) | [53] |
| 170 | [At1g73220](http://aramemnon.botanik.uni-koeln.de/seq_view.ep?orgm=0&search=At1g73220&cat=0&term=1) | Unknown | n.a. |
| 171 | [At1g14560](http://aramemnon.botanik.uni-koeln.de/seq_view.ep?orgm=0&search=At1g14560&cat=0&term=1) | putative mitochondrial carrier | n.a. |
| 172 | [At3g45040](http://aramemnon.botanik.uni-koeln.de/seq_view.ep?orgm=0&search=At3g45040&cat=0&term=1) | cytidylyltransferase family | n.a. |
| 173 | [At4g25640](http://aramemnon.botanik.uni-koeln.de/seq_view.ep?orgm=0&search=At4g25640&cat=0&term=1) | putative MATE-related efflux carrier (AtDTX35) | [3] |
| 174 | [At3g01550](http://aramemnon.botanik.uni-koeln.de/seq_view.ep?orgm=0&search=At3g01550&cat=0&term=1) | phosphate/phosphoenolpyruvate translocator (AtPPT2) | [33]* |
| 175 | [At1g70940](http://aramemnon.botanik.uni-koeln.de/seq_view.ep?orgm=0&search=At1g70940&cat=0&term=1) | auxin transporter (AtPIN3) | [54]* |
| 176 | [At3g53940](http://aramemnon.botanik.uni-koeln.de/seq_view.ep?orgm=0&search=At3g53940&cat=0&term=1) | putative mitochondrial carrier | n.a. |
| 177 | [At1g54730](http://aramemnon.botanik.uni-koeln.de/seq_view.ep?orgm=0&search=At1g54730&cat=0&term=1) | sugar transporter, putative | n.a. |
| 178 | [At1g79410](http://aramemnon.botanik.uni-koeln.de/seq_view.ep?orgm=0&search=At1g79410&cat=0&term=1) | Unknown | n.a. |
| 179 | [At4g38640](http://aramemnon.botanik.uni-koeln.de/seq_view.ep?orgm=0&search=At4g38640&cat=0&term=1) | choline transporter-related | n.a. |
| 180 | [At1g33110](http://aramemnon.botanik.uni-koeln.de/seq_view.ep?orgm=0&search=At1g33110&cat=0&term=1) | putative MATE-related efflux carrier (AtDTX21) | [3] |
| 181 | [At4g31600](http://aramemnon.botanik.uni-koeln.de/seq_view.ep?orgm=0&search=At4g31600&cat=0&term=1) | putative phosphate translocator-homolog, KT-group | [48] |
| 182 | [At1g15170](http://aramemnon.botanik.uni-koeln.de/seq_view.ep?orgm=0&search=At1g15170&cat=0&term=1) | putative MATE-related efflux carrier (AtDTX12) | [3] |
| 183 | [At5g24380](http://aramemnon.botanik.uni-koeln.de/seq_view.ep?orgm=0&search=At5g24380&cat=0&term=1) | nicotianamine-metal complex transporter, Yellow-Stripe-Like-2 (AtYSL2) | [4] |
| 184 | [At3g08040](http://aramemnon.botanik.uni-koeln.de/seq_view.ep?orgm=0&search=At3g08040&cat=0&term=1) | MATE-related efflux carrier, ferric reductase deficient (AtFRD3/AtDTX43) | [55] |
| 185 | [At4g32140](http://aramemnon.botanik.uni-koeln.de/seq_view.ep?orgm=0&search=At4g32140&cat=0&term=1) | Unknown | n.a. |
| 186 | [At2g34960](http://aramemnon.botanik.uni-koeln.de/seq_view.ep?orgm=0&search=At2g34960&cat=0&term=1) | putative cationic amino acid transporter (AtCAT5) | [12]* |
| 187 | [At1g08930](http://aramemnon.botanik.uni-koeln.de/seq_view.ep?orgm=0&search=At1g08930&cat=0&term=1) | putative sugar transporter, early dehydration induced protein (AtERD6) | [56]* |
| 188 | [At5g41800](http://aramemnon.botanik.uni-koeln.de/seq_view.ep?orgm=0&search=At5g41800&cat=0&term=1) | amino acid transporter family | n.a. |
| 189 | [At1g11670](http://aramemnon.botanik.uni-koeln.de/seq_view.ep?orgm=0&search=At1g11670&cat=0&term=1) | putative MATE-related efflux carrier (AtDTX36) | [3] |
| 190 | [At5g61520](http://aramemnon.botanik.uni-koeln.de/seq_view.ep?orgm=0&search=At5g61520&cat=0&term=1) | monosaccharide-proton symporter, green-leaf-specific (AtSTP3) | [57]* |
| 191 | [At3g21390](http://aramemnon.botanik.uni-koeln.de/seq_view.ep?orgm=0&search=At3g21390&cat=0&term=1) | putative mitochondrial carrier | n.a. |
| 192 | [At3g03090](http://aramemnon.botanik.uni-koeln.de/seq_view.ep?orgm=0&search=At3g03090&cat=0&term=1) | sugar transporter family | n.a. |
| 193 | [At3g59340](http://aramemnon.botanik.uni-koeln.de/seq_view.ep?orgm=0&search=At3g59340&cat=0&term=1) | hypothetical protein | n.a. |
| 194 | [At5g12860](http://aramemnon.botanik.uni-koeln.de/seq_view.ep?orgm=0&search=At5g12860&cat=0&term=1) | plastidic 2-oxoglutarate/malate translocator (AtDiT1/AtpOMT1) | [36]* |
| 195 | [At4g16370](http://aramemnon.botanik.uni-koeln.de/seq_view.ep?orgm=0&search=At4g16370&cat=0&term=1) | oligopeptide transporter (AtOPT3) | [58]* |
| 196 | [At3g55740](http://aramemnon.botanik.uni-koeln.de/seq_view.ep?orgm=0&search=At3g55740&cat=0&term=1) | proline transporter (AtProT2) | [59]* |
| 197 | [At1g31830](http://aramemnon.botanik.uni-koeln.de/seq_view.ep?orgm=0&search=At1g31830&cat=0&term=1) | putative neutral amino acid transport protein (AtLAT4) | n.a. |
| 198 | [At4g02050](http://aramemnon.botanik.uni-koeln.de/seq_view.ep?orgm=0&search=At4g02050&cat=0&term=1) | putative monosaccharide-proton symporter (AtSTP7) | [60] |
| 199 | [At5g44370](http://aramemnon.botanik.uni-koeln.de/seq_view.ep?orgm=0&search=At5g44370&cat=0&term=1) | putative anion transporter (AtANTR5) | n.a. |
| 200 | [At2g02020](http://aramemnon.botanik.uni-koeln.de/seq_view.ep?orgm=0&search=At2g02020&cat=0&term=1) | proton-dependent oligopeptide transport (POT) protein | n.a. |
| 201 | [At4g22790](http://aramemnon.botanik.uni-koeln.de/seq_view.ep?orgm=0&search=At4g22790&cat=0&term=1) | putative MATE-related efflux carrier (AtDTX56) | [3] |
| 202 | [At1g65730](http://aramemnon.botanik.uni-koeln.de/seq_view.ep?orgm=0&search=At1g65730&cat=0&term=1) | putative Fe(III)-phytosiderophore uptake mediator (AtYSL7) | [4] |
| 203 | [At5g01180](http://aramemnon.botanik.uni-koeln.de/seq_view.ep?orgm=0&search=At5g01180&cat=0&term=1) | proton-dependent oligopeptide transport (POT) protein | n.a. |
| 204 | [At5g01990](http://aramemnon.botanik.uni-koeln.de/seq_view.ep?orgm=0&search=At5g01990&cat=0&term=1) | auxin efflux carrier protein | n.a. |
| 205 | [At3g54510](http://aramemnon.botanik.uni-koeln.de/seq_view.ep?orgm=0&search=At3g54510&cat=0&term=1) | ERD4 protein-related | n.a. |
| 206 | [At1g62280](http://aramemnon.botanik.uni-koeln.de/seq_view.ep?orgm=0&search=At1g62280&cat=0&term=1) | C4-dicarboxylate transporter/malic acid transport protein | n.a. |
| 207 | [At4g35180](http://aramemnon.botanik.uni-koeln.de/seq_view.ep?orgm=0&search=At4g35180&cat=0&term=1) | putative lysine/histidine transporter (AtLHT7) | [7] |
| 208 | [At2g36590](http://aramemnon.botanik.uni-koeln.de/seq_view.ep?orgm=0&search=At2g36590&cat=0&term=1) | putative proline transporter (AtProT3) | [59] |
| 209 | [At2g38330](http://aramemnon.botanik.uni-koeln.de/seq_view.ep?orgm=0&search=At2g38330&cat=0&term=1) | putative MATE-related efflux carrier (AtDTX44) | [3] |
| 210 | [At1g30840](http://aramemnon.botanik.uni-koeln.de/seq_view.ep?orgm=0&search=At1g30840&cat=0&term=1) | putative purine permease (AtPUP10) | [61] |
| 211 | [At5g41760](http://aramemnon.botanik.uni-koeln.de/seq_view.ep?orgm=0&search=At5g41760&cat=0&term=1) | nucleotide-sugar transporter family | n.a. |
| 212 | [At4g16480](http://aramemnon.botanik.uni-koeln.de/seq_view.ep?orgm=0&search=At4g16480&cat=0&term=1) | putative inositol/polyol (cyclic)-proton symporter (AtINT4) | [43] |
| 213 | [At2g28780](http://aramemnon.botanik.uni-koeln.de/seq_view.ep?orgm=0&search=At2g28780&cat=0&term=1) | Unknown | n.a. |
| 214 | [At3g45700](http://aramemnon.botanik.uni-koeln.de/seq_view.ep?orgm=0&search=At3g45700&cat=0&term=1) | proton-dependent oligopeptide transport (POT) protein | n.a. |
| 215 | [At1g12500](http://aramemnon.botanik.uni-koeln.de/seq_view.ep?orgm=0&search=At1g12500&cat=0&term=1) | putative phosphate translocator-homolog, KV/A/G-group | [48] |
| 216 | [At3g07080](http://aramemnon.botanik.uni-koeln.de/seq_view.ep?orgm=0&search=At3g07080&cat=0&term=1) protein | Unknown | n.a. |
| 217 | [At1g28230](http://aramemnon.botanik.uni-koeln.de/seq_view.ep?orgm=0&search=At1g28230&cat=0&term=1) | purine permease (AtPUP1) | [61]* |
| 218 | [At5g36940](http://aramemnon.botanik.uni-koeln.de/seq_view.ep?orgm=0&search=At5g36940&cat=0&term=1) | putative cationic amino acid transporter (AtCAT3) | [12]* |
| 219 | [At2g43240](http://aramemnon.botanik.uni-koeln.de/seq_view.ep?orgm=0&search=At2g43240&cat=0&term=1) | nucleotide-sugar transporter family | n.a. |
| 220 | [At5g52050](http://aramemnon.botanik.uni-koeln.de/seq_view.ep?orgm=0&search=At5g52050&cat=0&term=1) | putative MATE-related efflux carrier (AtDTX50) | n.a. |
| 221 | [At4g36670](http://aramemnon.botanik.uni-koeln.de/seq_view.ep?orgm=0&search=At4g36670&cat=0&term=1) | putative polyol (linear)-proton symporter (AtPLT6) | [52] |
| 222 | [At4g21680](http://aramemnon.botanik.uni-koeln.de/seq_view.ep?orgm=0&search=At4g21680&cat=0&term=1) | proton-dependent oligopeptide transport (POT) protein | n.a. |
| 223 | [At2g04080](http://aramemnon.botanik.uni-koeln.de/seq_view.ep?orgm=0&search=At2g04080&cat=0&term=1) | putative MATE-related efflux carrier (AtDTX2) | [3] |
| 224 | [At4g04750](http://aramemnon.botanik.uni-koeln.de/seq_view.ep?orgm=0&search=At4g04750&cat=0&term=1) | sugar transporter family | n.a. |
| 225 | [At4g34100](http://aramemnon.botanik.uni-koeln.de/seq_view.ep?orgm=0&search=At4g34100&cat=0&term=1) | RING-domain protein with zinc-binding domain | n.a. |
| 226 | [At1g34580](http://aramemnon.botanik.uni-koeln.de/seq_view.ep?orgm=0&search=At1g34580&cat=0&term=1) | putative monosaccharide-proton symporter (AtSTP5) | [60] |
| 227 | [At4g38050](http://aramemnon.botanik.uni-koeln.de/seq_view.ep?orgm=0&search=At4g38050&cat=0&term=1) | putative nucleobase ascorbate transporter (AtNAT11) | n.a. |
| 228 | [At1g57980](http://aramemnon.botanik.uni-koeln.de/seq_view.ep?orgm=0&search=At1g57980&cat=0&term=1) | putative purine permease | n.a. |
| 229 | [At1g77210](http://aramemnon.botanik.uni-koeln.de/seq_view.ep?orgm=0&search=At1g77210&cat=0&term=1) | putative monosaccharide-proton symporter (AtSTP14) | n.a. |
| 230 | [At5g05630](http://aramemnon.botanik.uni-koeln.de/seq_view.ep?orgm=0&search=At5g05630&cat=0&term=1) | putative neutral amino acid transport protein (AtLAT1) | n.a. |
| 231 | [At4g18210](http://aramemnon.botanik.uni-koeln.de/seq_view.ep?orgm=0&search=At4g18210&cat=0&term=1) | putative purine permease (AtPUP7) | [14] |
| 232 | [At5g59740](http://aramemnon.botanik.uni-koeln.de/seq_view.ep?orgm=0&search=At5g59740&cat=0&term=1) | Unknown | n.a. |
| 233 | [At1g72140](http://aramemnon.botanik.uni-koeln.de/seq_view.ep?orgm=0&search=At1g72140&cat=0&term=1) | proton-dependent oligopeptide transport (POT) protein | n.a. |
| 234 | [At4g05120](http://aramemnon.botanik.uni-koeln.de/seq_view.ep?orgm=0&search=At4g05120&cat=0&term=1) | putative equilibrative nucleoside transporter (AtENT3) | [62] |
| 235 | [At5g23270](http://aramemnon.botanik.uni-koeln.de/seq_view.ep?orgm=0&search=At5g23270&cat=0&term=1) | pollen tube-specific monosaccharide-proton symporter (AtSTP11) | [63] |
| 236 | [At1g58340](http://aramemnon.botanik.uni-koeln.de/seq_view.ep?orgm=0&search=At1g58340&cat=0&term=1) | putative MATE-related efflux carrier (AtDTX48) | [3] |
| 237 | [At2g16990](http://aramemnon.botanik.uni-koeln.de/seq_view.ep?orgm=0&search=At2g16990&cat=0&term=1) | putative tetracycline transporter protein | n.a. |
| 238 | [At3g20660](http://aramemnon.botanik.uni-koeln.de/seq_view.ep?orgm=0&search=At3g20660&cat=0&term=1) | organic cation transporter family | n.a. |
| 239 | [At1g66570](http://aramemnon.botanik.uni-koeln.de/seq_view.ep?orgm=0&search=At1g66570&cat=0&term=1) | putative sucrose-proton symporter, pseudogene (AtSUC7) | [64] |

Reference List

1. Li Q, Schultes NP: **Arabidopsis thaliana locus At5g62890, a nucleobase-ascorbate transporter family member, is preferentially expressed in carpel transmitting tract and tapetal cells.** *Plant Science* 2002, **163:**233-240.

2. Klaus SMJ, Kunji ERS, Bozzo GG, Noiriel A, de la Garza RD, Basset GJC, Ravanel S, Rebeille F, Gregory JF, III, Hanson AD: **Higher Plant Plastids and Cyanobacteria Have Folate Carriers Related to Those of Trypanosomatids.** *J Biol Chem* 2005, **280:**38457-38463.

3. Li L, He Z, Pandey GK, Tsuchiya T, Luan S: **Functional Cloning and Characterization of a Plant Efflux Carrier for Multidrug and Heavy Metal Detoxification.** *J Biol Chem* 2002, **277:**5360-5368.

4. Curie C, Panaviene Z, Loulergue C, Dellaporta SL, Briat JF, Walker EL: **Maize yellow stripe1 encodes a membrane protein directly involved in Fe(III) uptake.** *Nature* 2001, **409:**346-349.

5. Koh S, Wiles AM, Sharp JS, Naider FR, Becker JM, Stacey G: **An Oligopeptide Transporter Gene Family in Arabidopsis.** *Plant Physiol* 2002, **128:**21-29.

6. Truernit E, Schmid J, Epple P, Illig J, Sauer N: **The Sink-Specific and Stress-Regulated Arabidopsis STP4 Gene: Enhanced Expression of a Gene Encoding a Monosaccharide Transporter by Wounding, Elicitors, and Pathogen Challenge.** *PLANT CELL* 1996, **8:**2169.

7. Hirner A, Ladwig F, Stransky H, Okumoto S, Keinath M, Harms A, Frommer WB, Koch W: **Arabidopsis LHT1 Is a High-Affinity Transporter for Cellular Amino Acid Uptake in Both Root Epidermis and Leaf Mesophyll.** *THE PLANT CELL* 2006, **18:**1931-1946.

8. Eicks M, Maurino V, Knappe S, Flugge UI, Fischer K: **The Plastidic Pentose Phosphate Translocator Represents a Link between the Cytosolic and the Plastidic Pentose Phosphate Pathways in Plants.** *Plant Physiol* 2002, **128:**512-522.

9. Song W, Steiner HY, Zhang L, Naider F, Stacey G, Becker JM: **Cloning of a Second Arabidopsis Peptide Transport Gene.** *Plant Physiol* 1996, **110:**171.

10. Bakker H, Routier F, Oelmann S, Jordi W, Lommen A, Gerardy-Schahn R, Bosch D: **Molecular cloning of two Arabidopsis UDP-galactose transporters by complementation of a deficient Chinese hamster ovary cell line.** *Glycobiology* 2005, **15:**193-201.

11. Dietrich D, Hammes U, Thor K, Suter-Grotemeyer M, Fluckiger R, Slusarenko AJ, Ward JM, Rentsch D: **AtPTR1, a plasma membrane peptide transporter expressed during seed germination and in vascular tissue of Arabidopsis.** *Plant J* 2004, **40:**488-499.

12. Su YH, Frommer WB, Ludewig U: **Molecular and Functional Characterization of a Family of Amino Acid Transporters from Arabidopsis.** *Plant Physiol* 2004, **136:**3104-3113.

13. Marchant A, Bhalerao R, Casimiro I, Eklof J, Casero PJ, Bennett M, Sandberg G: **AUX1 Promotes Lateral Root Formation by Facilitating Indole-3-Acetic Acid Distribution between Sink and Source Tissues in the Arabidopsis Seedling.** *THE PLANT CELL* 2002, **14:**589-597.

14. Gillissen B, Burkle L, Andre B, Kuhn C, Rentsch D, Brandl B, Frommer WB: **A New Family of High-Affinity Transporters for Adenine, Cytosine, and Purine Derivatives in Arabidopsis.** *THE PLANT CELL* 2000, **12:**291-300.

15. Quirino BF, Reiter WD, Amasino RD: **One of two tandem Arabidopsis genes homologous to monosaccharide transporters is senescence-associated.** *Plant Molecular Biology* 2001, **46:**447-457.

16. Norholm MHH, Nour-Eldin HH, Brodersen P, Mundy J, Halkier BA: **Expression of the Arabidopsis high-affinity hexose transporter STP13 correlates with programmed cell death.** *FEBS Letters* 2006, **580:**2381-2387.

17. Okumoto S, Schmidt R, Tegeder M, Fischer WN, Rentsch D, Frommer WB, Koch W: **High Affinity Amino Acid Transporters Specifically Expressed in Xylem Parenchyma and Developing Seeds of Arabidopsis.** *J Biol Chem* 2002, **277:**45338-45346.

18. Huang NC, Liu KH, Lo HJ, Tsay YF: **Cloning and Functional Characterization of an Arabidopsis Nitrate Transporter Gene That Encodes a Constitutive Component of Low-Affinity Uptake.** *THE PLANT CELL* 1999, **11:**1381.

19. Roth C, Menzel G, Petetot JM, Rochat-Hacker S, Poirier Y: **Characterization of a protein of the plastid inner envelope having homology to animal inorganic phosphate, chloride and organic-anion transporters.** *Planta* 2004, **218:**406-416.

20. Stadler R, Truernit E, Gahrtz M, Sauer N: **The AtSUC1 sucrose carrier may represent the osmotic driving force for anther dehiscence and pollen tube growth in Arabidopsis.** *The Plant Journal* 1999, **19:**269-278.

21. Blilou I, Xu J, Wildwater M, Willemsen V, Paponov I, Friml J, Heidstra R, Aida M, Palme K, Scheres B: **The PIN auxin efflux facilitator network controls growth and patterning in Arabidopsis roots.** *Nature* 2005, **433:**39-44.

22. Chandran D, Reinders A, Ward JM: **Substrate Specificity of the Arabidopsis thaliana Sucrose Transporter AtSUC2.** *J Biol Chem* 2003, **278:**44320-44325.

23. Waterworth WM, West CE, Bray CM: **The barley scutellar peptide transporter: biochemical characterization and localization to the plasma membrane.** *J Exp Bot* 2000, **51:**1201-1209.

24. Weber A, Servaites JC, Geiger DR, Kofler H, Hille D, Groner F, Hebbeker U, Flugge UI: **Identification, Purification, and Molecular Cloning of a Putative Plastidic Glucose Translocator.** *THE PLANT CELL* 2000, **12:**787-802.

25. Fischer WN, Kwart M, Hummel S, Frommer WB: **Substrate Specificity and Expression Profile of Amino Acid Transporters (AAPs) in Arabidopsis.** *J Biol Chem* 1995, **270:**16315.

26. Sherson SM, Hemmann G, Wallace G, Forbes S, Germain V, Stadler R, Bechtold N, Sauer N, Smith SM: **Monosaccharide/proton symporter AtSTP1 plays a major role in uptake and response of Arabidopsis seeds and seedlings to sugars.** *The Plant Journal* 2000, **24:**849-857.

27. Karim S, Lundh D, Holmstrom KO, Mandal A, Pirhonen M: **Structural and functional characterization of AtPTR3, a stress-induced peptide transporter of Arabidopsis.** *J Mol Model (Online)* 2005, **11:**226-236.

28. Niewiadomski P, Knappe S, Geimer S, Fischer K, Schulz B, Unte US, Rosso MG, Ache P, Flugge UI, Schneider A: **The Arabidopsis Plastidic Glucose 6-Phosphate/Phosphate Translocator GPT1 Is Essential for Pollen Maturation and Embryo Sac Development.** *THE PLANT CELL* 2005, **17:**760-775.

29. Reiser J, Linka N, Lemke L, Jeblick W, Neuhaus HE: **Molecular Physiological Analysis of the Two Plastidic ATP/ADP Transporters from Arabidopsis.** *Plant Physiol* 2004, **136:**3524-3536.

30. Weise A, Barker L, Kuhn C, Lalonde S, Buschmann H, Frommer WB, Ward JM: **A New Subfamily of Sucrose Transporters, SUT4, with Low Affinity/High Capacity Localized in Enucleate Sieve Elements of Plants.** *THE PLANT CELL* 2000, **12:**1345.

31. Lasswell J, Rogg LE, Nelson DC, Rongey C, Bartel B: **Cloning and Characterization of IAR1, a Gene Required for Auxin Conjugate Sensitivity in Arabidopsis.** *THE PLANT CELL* 2000, **12:**2395.

32. Mohlmann T, Mezher Z, Schwerdtfeger G, Neuhaus HE: **Characterisation of a concentrative type of adenosine transporter from Arabidopsis thaliana (ENT1,At).** *FEBS Letters* 2001, **509:**370-374.

33. Knappe S, Lottgert T, Schneider A, Voll L, Flugge UI, Fischer K: **Characterization of two functional phosphoenolpyruvate/phosphate translocator (PPT) genes in Arabidopsis-AtPPT1 may be involved in the provision of signals for correct mesophyll development.** *The Plant Journal* 2003, **36:**411-420.

34. Kwart M, Hirner B, Hummel S, Frommer WB: **Differential expression of two related amino acid transporters with differing substrate specificity in Arabidopsis thaliana.** *The Plant Journal* 1993, **4:**993-1002.

35. Desimone M, Catoni E, Ludewig U, Hilpert M, Schneider A, Kunze R, Tegeder M, Frommer WB, Schumacher K: **A Novel Superfamily of Transporters for Allantoin and Other Oxo Derivatives of Nitrogen Heterocyclic Compounds in Arabidopsis.** *THE PLANT CELL* 2002, **14:**847-856.

36. Taniguchi M, Taniguchi Y, Kawasaki M, Takeda S, Kato T, Sato S, Tabata S, Miyake H, Sugiyama T: **Identifying and Characterizing Plastidic 2-Oxoglutarate/Malate and Dicarboxylate Transporters in Arabidopsis thaliana.** *Plant Cell Physiol* 2002, **43:**706-717.

37. Wang HX, Weerasinghe RR, Perdue TD, Cakmakci NG, Taylor JP, Marzluff WF, Jones AM: **A Golgi-localized Hexose Transporter Is Involved in Heterotrimeric G Protein-mediated Early Development in Arabidopsis.** *Mol Biol Cell* 2006,E06-01.

38. Schaaf G, Honsbein A, Meda AR, Kirchner S, Wipf D, von Wiren N: **AtIREG2 Encodes a Tonoplast Transport Protein Involved in Iron-dependent Nickel Detoxification in Arabidopsis thaliana Roots.** *J Biol Chem* 2006, **281:**25532-25540.

39. Saier MH, Jr.: **A Functional-Phylogenetic Classification System for Transmembrane Solute Transporters.** *Microbiol Mol Biol Rev* 2000, **64:**354-411.

40. Friml J, Benkova E, Blilou I, Wisniewska J, Hamann T, Ljung K, Woody S, Sandberg G, Scheres B, Jurgens G et al.: **AtPIN4 Mediates Sink-Driven Auxin Gradients and Root Patterning in Arabidopsis.** *Cell* 2002, **108:**661-673.

41. Fischer WN, Loo DDF, Koch W, Ludewig U, Boorer KJ, Tegeder M, Rentsch D, Wright EM, Frommer WB: **Low and high affinity amino acid H+-cotransporters for cellular import of neutral and charged amino acids.** *The Plant Journal* 2002, **29:**717-731.

42. Muller A, Guan C, Galweiler L, Tanzler P, Huijser P, Marchant A, Parry G, Bennett M, Wisman E, Palme K: **AtPIN2 defines a locus of Arabidopsis for root gravitropism control.** *EMBO J* 1998, **17:**6903-6911.

43. Schneider S, Schneidereit A, Konrad KR, Hajirezaei MR, Gramann M, Hedrich R, Sauer N: **Arabidopsis INOSITOL TRANSPORTER4 Mediates High-Affinity H+ Symport of Myoinositol across the Plasma Membrane.** *Plant Physiol* 2006, **141:**565-577.

44. Okumoto S, Koch W, Tegeder M, Fischer WN, Biehl A, Leister D, Stierhof YD, Frommer WB: **Root phloem-specific expression of the plasma membrane amino acid proton co-transporter AAP3.** *J Exp Bot* 2004, **55:**2155-2168.

45. Chiu CC, Lin CS, Hsia AP, Su RC, Lin HL, Tsay YF: **Mutation of a Nitrate Transporter, AtNRT1:4, Results in a Reduced Petiole Nitrate Content and Altered Leaf Development.** *Plant Cell Physiol* 2004, **45:**1139-1148.

46. Osawa H, Stacey G, Gassmann W: **ScOPT1 and AtOPT4 function as proton-coupled oligopeptide transporters with broad but distinct substrate specificities.** *Biochem J* 2006, **393:**267-275.

47. Norambuena L, Marchant L, Berninsone P, Hirschberg CB, Silva H, Orellana A: **Transport of UDP-galactose in Plants. Identification and functional characterization of AtUTr1, an Arabidopsis thaliana UDP-Galactose/UDP-Glucose transporter.** *J Biol Chem* 2002, **277:**32923-32929.

48. Knappe S, Flugge UI, Fischer K: **Analysis of the Plastidic phosphate translocator Gene Family in Arabidopsis and Identification of New phosphate translocator-Homologous Transporters, Classified by Their Putative Substrate-Binding Site.** *Plant Physiol* 2003, **131:**1178-1190.

49. lweiler L, Guan C, ller A, Wisman E, Mendgen K, Yephremov A, Palme K: **Regulation of Polar Auxin Transport by AtPIN1 in Arabidopsis Vascular Tissue.** *Science* 1998, **282:**2226-2230.

50. Boorer KJ, Fischer WN: **Specificity and Stoichiometry of the Arabidopsis H+/Amino Acid Transporter AAP5.** *J Biol Chem* 1997, **272:**13040-13046.

51. Wang R, Liu D, Crawford NM: **The Arabidopsis CHL1 protein plays a major role in high-affinity nitrate uptake.** *PNAS* 1998, **95:**15134-15139.

52. Reinders A, Panshyshyn JA, Ward JM: **Analysis of Transport Activity of Arabidopsis Sugar Alcohol Permease Homolog AtPLT5.** *J Biol Chem* 2005, **280:**1594-1602.

53. Sanchez-Fernandez R, Davies TGE, Coleman JOD, Rea PA: **The Arabidopsis thaliana ABC Protein Superfamily, a Complete Inventory.** *J Biol Chem* 2001, **276:**30231-30244.

54. Friml J, Wisniewska J, Benkova E, Mendgen K, Palme K: **Lateral relocation of auxin efflux regulator PIN3 mediates tropism in Arabidopsis.** *Nature* 2002, **415:**806-809.

55. Rogers EE, Guerinot ML: **FRD3, a Member of the Multidrug and Toxin Efflux Family, Controls Iron Deficiency Responses in Arabidopsis.** *THE PLANT CELL* 2002, **14:**1787-1799.

56. Kiyosue T, Abe H, Yamaguchi-Shinozaki K, Shinozaki K: **ERD6, a cDNA clone for an early dehydration-induced gene of Arabidopsis, encodes a putative sugar transporter.** *Biochimica et Biophysica Acta (BBA) - Biomembranes* 1998, **1370:**187-191.

57. Buttner M, Truernit E, Baier K, Scholz-Starke J, Sontheim M, Lauterbach C, Huss VAR, Sauer N: **AtSTP3, a green leaf-specific, low affinity monosaccharide-H+ symporter of Arabidopsis thaliana.** *Plant, Cell and Environment* 2000, **23:**175-184.

58. Stacey MG, Koh S, Becker J, Stacey G: **AtOPT3, a Member of the Oligopeptide Transporter Family, Is Essential for Embryo Development in Arabidopsis.** *THE PLANT CELL* 2002, **14:**2799-2811.

59. Grallath S, Weimar T, Meyer A, Gumy C, Suter-Grotemeyer M, Neuhaus JM, Rentsch D: **The AtProT Family. Compatible Solute Transporters with Similar Substrate Specificity But Differential Expression Patterns.** *Plant Physiol* 2005, **137:**117-126.

60. Buttner M, Sauer N: **Monosaccharide transporters in plants: structure, function and physiology.** *Biochimica et Biophysica Acta (BBA) - Biomembranes* 2000, **1465:**263-274.

61. Burkle L, Cedzich A, Dopke C, Stransky H, Okumoto S, Gillissen B, Kuhn C, Frommer WB: **Transport of cytokinins mediated by purine transporters of the PUP family expressed in phloem, hydathodes, and pollen of Arabidopsis.** *The Plant Journal* 2003, **34:**13-26.

62. Chen KL, Xu MX, Li GY, Liang H, Xia ZL, Liu X, Zhang JS, Zhang AM, Wang DW: **Identification of AtENT3 as the main transporter for uridine uptake in Arabidopsis roots.** *Cell Res* 0 AD, **16:**377-388.

63. Schneidereit A, Scholz-Starke J, Sauer N, B++ttner M: **AtSTP11, a pollen tube-specific monosaccharide transporter in<i> Arabidopsis</i>.** *Planta* 2005, **221:**48-55.

64. Sauer N, Ludwig A, Knoblauch A, Rothe P, Gahrtz M, Klebl F: **AtSUC8 and AtSUC9 encode functional sucrose transporters, but the closely related AtSUC6 and AtSUC7 genes encode aberrant proteins in different Arabidopsis ecotypes.** *The Plant Journal* 2004, **40:**120-130.
