# Supplementary material for: Screening for plant transporter function by expressing a normalized Arabidopsis full-length cDNA library in Xenopus oocytes
Source: Plant Methods. 2006 Oct 27;2:17. doi: 10.1186/1746-4811-2-17 (PMC1637106; doi:10.1186/1746-4811-2-17)
Supplement: Additional File 2 — USER cloning primers. This table provides a list of the primers used to amplify the individual CDS'es from each full length cDNA. [file 1746-4811-2-17-S2.doc]

**Additional file 2**

Table 2 Primers used to amplify the individual CDS from each full length cDNA. The first 8 nucleotides of each primer are the uracil containing tails used to clone the PCR fragments into the *Xenopus* oocyte expression vector using the improved USER cloning technique [1]. Each AGI code is a hyperlink to its corresponding page on ARAMEMNON

Reference List

1. Nour-Eldin HH, Hansen BG, Norholm MHH, Jensen JK, Halkier BA: **Advancing uracil-excision based cloning towards an ideal technique for cloning PCR fragments.** *Nucl Acids Res* 2006, gkl635.

**Table 2**

| Nr. | AGI code | 5’ forward primers | 3´ reverse primers |
| --- | --- | --- | --- |
| 1 | [At2g34190](http://aramemnon.botanik.uni-koeln.de/seq_view.ep?x=0&y=0&search=At2g34190+) | GGCTTAAUATGGATCTTGTAAAGCCA | GGTTTAAUCTATGAAGGAGGGAAGAA |
| 2 | [At1g64890](http://aramemnon.botanik.uni-koeln.de/seq_view.ep?orgm=0&search=At1g64890&cat=0&term=1) | GGCTTAAUATGAGCAGCAGCAGCGAT | GGTTTAAUTCACTCTATCTTTTTACT |
| 3 | [AT5g65380](http://aramemnon.botanik.uni-koeln.de/seq_view.ep?orgm=0&search=AT5g65380&cat=0&term=1#bottom) | GGCTTAAUATGAGGGGAGGTGATGGA | GGTTTAAUTCATTTTATTGTGTTGGA |
| 4 | [At1g48370](http://aramemnon.botanik.uni-koeln.de/seq_view.ep?orgm=0&search=At1g48370&cat=0&term=1) | GGCTTAAUATGAGAAAAGGAGGTTTA | GGTTTAAUCTAAGAGGATCCTTGCAG |
| 5 | [At1g61890](http://aramemnon.botanik.uni-koeln.de/seq_view.ep?orgm=0&search=At1g61890&cat=0&term=1) | GGCTTAAUATGAATTCAGAATCGCTA | GGTTTAAUTTATTGCTTCAAAAGCGG |
| 6 | [AT4g10770](http://aramemnon.botanik.uni-koeln.de/seq_view.ep?orgm=0&search=AT4g10770&cat=0&term=1) | GGCTTAAUATGGAAGAATCAGAACAA | GGTTTAAUTTACGTATAAAGCGGGCA |
| 7 | [At3g19930](http://aramemnon.botanik.uni-koeln.de/seq_view.ep?orgm=0&search=At3g19930&cat=0&term=1) | GGCTTAAUATGGCCGGAGGGTTCGTC | GGTTTAAUTCATACGGACTTCTGTTG |
| 8 | [AT5g40780](http://aramemnon.botanik.uni-koeln.de/seq_view.ep?orgm=0&search=AT5g40780&cat=0&term=1) | GGCTTAAUATGGTAGCTCAAGCTCCT | GGTTTAAUTTATGAGTAAAACTTGTA |
| 9 | [At1g08920](http://aramemnon.botanik.uni-koeln.de/seq_view.ep?orgm=0&search=At1g08920&cat=0&term=1) | GGCTTAAUATGACGATGTCGGAGAAC | GGTTTAAUTCATTGTAGAAAATCTGT |
| 10 | [AT5g38030](http://aramemnon.botanik.uni-koeln.de/seq_view.ep?orgm=0&search=AT5g38030&cat=0&term=1) | GGCTTAAUATGGAAGAAGACAAAATT | GGTTTAAUTTAGTTTAAGAGTTGATC |
| 11 | [AT3g53960](http://aramemnon.botanik.uni-koeln.de/seq_view.ep?orgm=0&search=AT3g53960&cat=0&term=1) | GGCTTAAUATGCATGTAAGTGATTCC | GGTTTAAUTTACGTAAACTTGGACGT |
| 12 | [AT3g21670](http://aramemnon.botanik.uni-koeln.de/seq_view.ep?orgm=0&search=AT3g21670&cat=0&term=1) | GGCTTAAUATGGTTCATGTGTCATCA | GGTTTAAUTCAAGGAATGTCCTTAAG |
| 13 | [AT5g17630](http://aramemnon.botanik.uni-koeln.de/seq_view.ep?orgm=0&search=AT5g17630&cat=0&term=1) | GGCTTAAUATGATCTCCCTGAATCTA | GGTTTAAUTTAGTTCTTCTTATCACC |
| 14 | [At2g02040](http://aramemnon.botanik.uni-koeln.de/seq_view.ep?orgm=0&search=At2g02040&cat=0&term=1) | GGCTTAAUATGGGTTCCATCGAAGAA | GGTTTAAUCTACGACGAAGCTTTCTT |
| 15 | [At1g66760](http://aramemnon.botanik.uni-koeln.de/seq_view.ep?orgm=0&search=At1g66760&cat=0&term=1) | GGCTTAAUATGAAGAAGAGTATCGAA | GGTTTAAUTCAATTAAGCAATGAGTC |
| 16 | [AT3g47420](http://aramemnon.botanik.uni-koeln.de/seq_view.ep?orgm=0&search=AT3g47420&cat=0&term=1) | GGCTTAAUATGGGTTCTCTAATGCAA | GGTTTAAUTCACACTTCCATCACATG |
| 17 | [At1g77610](http://aramemnon.botanik.uni-koeln.de/seq_view.ep?orgm=0&search=At1g77610&cat=0&term=1) | GGCTTAAUATGGAGGAAGGAAGTATG | GGTTTAAUTCAGACTTTGCCTTCGAG |
| 18 | [At3g54140](http://aramemnon.botanik.uni-koeln.de/seq_view.ep?orgm=0&search=At3g54140&cat=0&term=1) | GGCTTAAUATGGAAGAAAAAGATGTG | GGTTTAAUTCAATGTGCTCGACCAAC |
| 19 | [At3g47960](http://aramemnon.botanik.uni-koeln.de/seq_view.ep?orgm=0&search=At3g47960&cat=0&term=1) | GGCTTAAUATGGAGAGAAAGCCTCTT | GGTTTAAUTCAGACAGAGTTCTTGTC |
| 20 | [At1g05940](http://aramemnon.botanik.uni-koeln.de/seq_view.ep?orgm=0&search=At1g05940&cat=0&term=1) | GGCTTAAUATGGGAGGCCACGAAGGT | GGTTTAAUCTAAGCGTCGCTTTCAGT |
| 21 | [At2g26510](http://aramemnon.botanik.uni-koeln.de/seq_view.ep?orgm=0&search=At2g26510&cat=0&term=1) | GGCTTAAUATGGTTGAAACTGGTCAC | GGTTTAAUTCAAAGGAACCGTGTCGG |
| 22 | [At2g21050](http://aramemnon.botanik.uni-koeln.de/seq_view.ep?orgm=0&search=At2g21050&cat=0&term=1) | GGCTTAAUATGGAGAACGGTGAGAAA | GGTTTAAUTCAAAGGCCGTGAGTGTG |
| 23 | [At5g55930](http://aramemnon.botanik.uni-koeln.de/seq_view.ep?orgm=0&search=At5g55930&cat=0&term=1) | GGCTTAAUATGACGAGCGTTTTCGAC | GGTTTAAUTTAAAACACGGGACAACC |
| 24 | [At4g12030](http://aramemnon.botanik.uni-koeln.de/seq_view.ep?orgm=0&search=At4g12030&cat=0&term=1) | GGCTTAAUATGATGTTTGCTGTTGGT | GGTTTAAUCTACTCCTTTCTGTTTTT |
| 25 | [At4g38250](http://aramemnon.botanik.uni-koeln.de/seq_view.ep?orgm=0&search=At4g38250&cat=0&term=1) | GGCTTAAUATGGGTTTTCAGAACGAA | GGTTTAAUTCACACTTTGACAGAGAA |
| 26 | [At1g44750](http://aramemnon.botanik.uni-koeln.de/seq_view.ep?orgm=0&search=At1g44750&cat=0&term=1) | GGCTTAAUATGGCGAAGGAACCAGTT | GGTTTAAUTCAACAGGGCGGTTCTAC |
| 27 | [At5g27350](http://aramemnon.botanik.uni-koeln.de/seq_view.ep?orgm=0&search=At5g27350&cat=0&term=1) | GGCTTAAUATGATTTATTTCTGTGGG | GGTTTAAUTTAAGTTTGATTTCTTTC |
| 28 | [At1g57990](http://aramemnon.botanik.uni-koeln.de/seq_view.ep?orgm=0&search=At1g57990&cat=0&term=1) | GGCTTAAUATGGAGATGACCGAAGCT | GGTTTAAUCTAAACGTCAATATTGTT |
| 29 | [At5g26340](http://aramemnon.botanik.uni-koeln.de/seq_view.ep?orgm=0&search=At5g26340&cat=0&term=1) | GGCTTAAUATGACCGGAGGAGGATTT | GGTTTAAUTTAAAGCCGTGTTGAAGG |
| 30 | [At1g75220](http://aramemnon.botanik.uni-koeln.de/seq_view.ep?orgm=0&search=At1g75220&cat=0&term=1) | GGCTTAAUATGAGTTTCAGGGATGAT | GGTTTAAUTCATCTGAACAAGGATTG |
| 31 | [At5g23810](http://aramemnon.botanik.uni-koeln.de/seq_view.ep?orgm=0&search=At5g23810&cat=0&term=1) | GGCTTAAUATGGTTTTGTTTGGTTTG | GGTTTAAUTCATCCGAATTTAGCTCC |
| 32 | [At1g47670](http://aramemnon.botanik.uni-koeln.de/seq_view.ep?orgm=0&search=At1g47670&cat=0&term=1) | GGCTTAAUATGGACGAAAGACCCGAG | GGTTTAAUTTAGTTAGGCGGCTTGAA |
| 33 | [At4g22840](http://aramemnon.botanik.uni-koeln.de/seq_view.ep?orgm=0&search=At4g22840&cat=0&term=1) | GGCTTAAUATGAGCGTGATCACAACT | GGTTTAAUTTAAAATGTGTTACTCTT |
| 34 | [At1g69850](http://aramemnon.botanik.uni-koeln.de/seq_view.ep?orgm=0&search=At1g69850&cat=0&term=1) | GGCTTAAUATGGAAGTGGAAGAAGAG | GGTTTAAUTTAGCTTCTTGAACCAGT |
| 35 | [At3g46980](http://aramemnon.botanik.uni-koeln.de/seq_view.ep?orgm=0&search=At3g46980&cat=0&term=1) | GGCTTAAUATGTGTTACTCTCTCTCT | GGTTTAAUTCAAGCTGTTGTGTCAAA |
| 36 | [At3g03720](http://aramemnon.botanik.uni-koeln.de/seq_view.ep?orgm=0&search=At3g03720&cat=0&term=1) | GGCTTAAUATGAACAGTCTTGTGAGA | GGTTTAAUTTAAGCAAGATGATCTGT |
| 37 | [At1g72120](http://aramemnon.botanik.uni-koeln.de/seq_view.ep?orgm=0&search=At1g72120&cat=0&term=1) | GGCTTAAUATGACGACTACTTCAGAA | GGTTTAAUCTACACTCGATCCACTCG |
| 38 | [At1g71880](http://aramemnon.botanik.uni-koeln.de/seq_view.ep?orgm=0&search=At1g71880&cat=0&term=1) | GGCTTAAUATGGGAGCCTATGAAACA | GGTTTAAUCTAGTGGAATCCTCCCAT |
| 39 | [At1g23080](http://aramemnon.botanik.uni-koeln.de/seq_view.ep?orgm=0&search=At1g23080&cat=0&term=1) | GGCTTAAUATGATCACATGGCACGAC | GGTTTAAUTTAACGGAAAAAAGAAAC |
| 40 | [At1g22710](http://aramemnon.botanik.uni-koeln.de/seq_view.ep?orgm=0&search=At1g22710&cat=0&term=1) | GGCTTAAUATGGTCAGCCATCCAATG | GGTTTAAUTCAATGAAATCCCATAGT |
| 41 | [At3g13620](http://aramemnon.botanik.uni-koeln.de/seq_view.ep?orgm=0&search=At3g13620&cat=0&term=1) | GGCTTAAUATGGCAATCTCAGAGGCA | GGTTTAAUTCATGAATTGTGATCATC |
| 42 | [At3g45680](http://aramemnon.botanik.uni-koeln.de/seq_view.ep?orgm=0&search=At3g45680&cat=0&term=1) | GGCTTAAUATGGCGGCGTTGATGGAG | GGTTTAAUTCAGAATGTGATGACTTG |
| 43 | [At2g29650](http://aramemnon.botanik.uni-koeln.de/seq_view.ep?orgm=0&search=At2g29650&cat=0&term=1) | GGCTTAAUATGAACGCGAGAGCTCTT | GGTTTAAUTCAATCGATTATCTTCTC |
| 44 | [At1g62200](http://aramemnon.botanik.uni-koeln.de/seq_view.ep?orgm=0&search=At1g62200&cat=0&term=1) | GGCTTAAUATGGTGAATTCGAATGAA | GGTTTAAUTTACAAAGCCTTCTTCTT |
| 45 | [At3g17650](http://aramemnon.botanik.uni-koeln.de/seq_view.ep?orgm=0&search=At3g17650&cat=0&term=1) | GGCTTAAUATGAGAAAGGGAGTTCTA | GGTTTAAUTTAAATGGATCCTTTCAG |
| 46 | [At1g47530](http://aramemnon.botanik.uni-koeln.de/seq_view.ep?orgm=0&search=At1g47530&cat=0&term=1) | GGCTTAAUATGGGAAAGGATAAGACT | GGTTTAAUTCACTCCTGCGCCGTTCC |
| 47 | [At5g16150](http://aramemnon.botanik.uni-koeln.de/seq_view.ep?orgm=0&search=At5g16150&cat=0&term=1) | GGCTTAAUATGCAGTCGTCAACGTAT | GGTTTAAUTCAAGCTCCAGATGTAAG |
| 48 | [At1g58360](http://aramemnon.botanik.uni-koeln.de/seq_view.ep?orgm=0&search=At1g58360&cat=0&term=1) | GGCTTAAUATGAAGAGTTTCAACACA | GGTTTAAUTCACTCATGCATAGTCCG |
| 49 | [At1g49960](http://aramemnon.botanik.uni-koeln.de/seq_view.ep?orgm=0&search=At1g49960&cat=0&term=1) | GGCTTAAUATGGCAACAAAGACCGAT | GGTTTAAUTCAGAATGAAGGGAAGAA |
| 50 | [At1g77690](http://aramemnon.botanik.uni-koeln.de/seq_view.ep?orgm=0&search=At1g77690&cat=0&term=1) | GGCTTAAUATGGCGGCAGAGAAAATA | GGTTTAAUTCATGGCTTGTGAGGAGG |
| 51 | [At4g21910](http://aramemnon.botanik.uni-koeln.de/seq_view.ep?orgm=0&search=At4g21910&cat=0&term=1) | GGCTTAAUATGGATGTGTCAAATGAG | GGTTTAAUCTAGTTTTGGAGAGGCTC |
| 52 | [At1g11260](http://aramemnon.botanik.uni-koeln.de/seq_view.ep?orgm=0&search=At1g11260&cat=0&term=1) | GGCTTAAUATGCCTGCCGGTGGATTC | GGTTTAAUTCAAACATGCTTCGTTCC |
| 53 | [At2g25520](http://aramemnon.botanik.uni-koeln.de/seq_view.ep?orgm=0&search=At2g25520&cat=0&term=1) | GGCTTAAUATGGGGAAAGGTCGTGCA | GGTTTAAUTCAATCTTGGGTTTCGTT |
| 54 | [At5g18840](http://aramemnon.botanik.uni-koeln.de/seq_view.ep?orgm=0&search=At5g18840&cat=0&term=1) | GGCTTAAUATGGCCATTAGGGAAATC | GGTTTAAUTTATGTTTCTCTTCGAAT |
| 55 | [At1g69870](http://aramemnon.botanik.uni-koeln.de/seq_view.ep?orgm=0&search=At1g69870&cat=0&term=1) | GGCTTAAUATGGTTTTGGAGGATAGA | GGTTTAAUTCAATAAGTTAAATAAAA |
| 56 | [At5g46050](http://aramemnon.botanik.uni-koeln.de/seq_view.ep?orgm=0&search=At5g46050&cat=0&term=1) | GGCTTAAUATGACAGTAGAAGAGGTA | GGTTTAAUTTATTCAGTCTCTTTCAT |
| 57 | [At2g40460](http://aramemnon.botanik.uni-koeln.de/seq_view.ep?orgm=0&search=At2g40460&cat=0&term=1) | GGCTTAAUATGGAGGCTGCAAAAGTT | GGTTTAAUTTAGATACTAAGAGGAGA |
| 58 | [At1g17120](http://aramemnon.botanik.uni-koeln.de/seq_view.ep?orgm=0&search=At1g17120&cat=0&term=1) | GGCTTAAUATGATCCCTGCTTCAATG | GGTTTAAUTTATCTCTCTCCCTCAAA |
| 59 | [At1g18880](http://aramemnon.botanik.uni-koeln.de/seq_view.ep?orgm=1&search=At1g18880&cat=0&term=1) | GGCTTAAUATGGAGGTTGAGAAGACA | GGTTTAAUTTACACTGACACCTTATC |
| 60 | [At3g26590](http://aramemnon.botanik.uni-koeln.de/seq_view.ep?orgm=0&search=At3g26590&cat=0&term=1) | GGCTTAAUATGGCAAAAGACAAAGAT | GGTTTAAUTTAGTTTATGAGTTGCTT |
| 61 | [At5g54800](http://aramemnon.botanik.uni-koeln.de/seq_view.ep?orgm=0&search=At5g54800&cat=0&term=1) | GGCTTAAUATGGTTTTATCGGTGAAG | GGTTTAAUTCAGAGCTTTGCCTGGGA |
| 62 | [At1g80300](http://aramemnon.botanik.uni-koeln.de/seq_view.ep?orgm=0&search=At1g80300&cat=0&term=1) | GGCTTAAUATGGAAGCTGTGATTCAA | GGTTTAAUTTATAAGTTGGTGGGAGC |
| 63 | [At1g15150](http://aramemnon.botanik.uni-koeln.de/seq_view.ep?orgm=0&search=At1g15150&cat=0&term=1) | GGCTTAAUATGCAAGACGCGGAGAGA | GGTTTAAUTTAGATGGGAAGTTCTGA |
| 64 | [At1g09960](http://aramemnon.botanik.uni-koeln.de/seq_view.ep?orgm=0&search=At1g09960&cat=0&term=1) | GGCTTAAUATGGCTACTTCCGATCAA | GGTTTAAUTCATGGGAGAGGGATGGG |
| 65 | [At3g30390](http://aramemnon.botanik.uni-koeln.de/seq_view.ep?orgm=0&search=At3g30390&cat=0&term=1) | GGCTTAAUATGACAGTTGTTGGAGAT | GGTTTAAUTTACTCACGAGGAGCGTT |
| 66 | [At1g80510](http://aramemnon.botanik.uni-koeln.de/seq_view.ep?orgm=0&search=At1g80510&cat=0&term=1) | GGCTTAAUATGGATAGCAGTTACTCT | GGTTTAAUTCAATCTGATTTGCTCTC |
| 67 | [At1g68100](http://aramemnon.botanik.uni-koeln.de/seq_view.ep?orgm=0&search=At1g68100&cat=0&term=1) | GGCTTAAUATGTCGTTCTCGCTGAGA | GGTTTAAUTCAGTGAAATTGTGTTAC |
| 68 | [At1g16390](http://aramemnon.botanik.uni-koeln.de/seq_view.ep?orgm=0&search=At1g16390&cat=0&term=1) | GGCTTAAUATGGCCGACTCGACTCGG | GGTTTAAUTCAACCAATAAATTGTCT |
| 69 | [At3g48200](http://aramemnon.botanik.uni-koeln.de/seq_view.ep?orgm=0&search=At3g48200&cat=0&term=1) | GGCTTAAUATGGGTCTGCTTAAAACT | GGTTTAAUTTACTTTGAAGCAAAAAT |
| 70 | [At1g32080](http://aramemnon.botanik.uni-koeln.de/seq_view.ep?orgm=0&search=At1g32080&cat=0&term=1) | GGCTTAAUATGGCTACTCTTTTAGCC | GGTTTAAUTCAGCCGACGACCGCTAG |
| 71 | [At5g27730](http://aramemnon.botanik.uni-koeln.de/seq_view.ep?orgm=0&search=At5g27730&cat=0&term=1) | GGCTTAAUATGGCGGAAATCAAAGTG | GGTTTAAUTTAGAGTTTCCAATAGAT |
| 72 | [At5g64500](http://aramemnon.botanik.uni-koeln.de/seq_view.ep?orgm=0&search=At5g64500&cat=0&term=1) | GGCTTAAUATGGATGTTGACGGAGAA | GGTTTAAUTCATGCTTCCTGGAGAAG |
| 73 | [At5g54860](http://aramemnon.botanik.uni-koeln.de/seq_view.ep?orgm=0&search=At5g64500&cat=0&term=1) | GGCTTAAUATGATACATTGGTTGAAG | GGTTTAAUCTATGATGCTGATACTCC |
| 74 | [At1g70330](http://aramemnon.botanik.uni-koeln.de/seq_view.ep?orgm=0&search=At1g70330&cat=0&term=1) | GGCTTAAUATGACTCCCATTAGTCAA | GGTTTAAUTCAAATGACCCAGAACCA |
| 75 | [At1g71090](http://aramemnon.botanik.uni-koeln.de/seq_view.ep?orgm=0&search=At1g71090&cat=0&term=1) | GGCTTAAUATGTCAGGTTTCTCCAGT | GGTTTAAUTTATTGCATACCTTGGAC |
| 76 | [At4g35870](http://aramemnon.botanik.uni-koeln.de/seq_view.ep?orgm=0&search=At4g35870&cat=0&term=1) | GGCTTAAUATGAATCGCAATTTTTCA | GGTTTAAUTCATCTGTTGTTGTAAGT |
| 77 | [At5g33320](http://aramemnon.botanik.uni-koeln.de/seq_view.ep?orgm=0&search=At5g33320&cat=0&term=1) | GGCTTAAUATGCAAAGCTCCGCCGTA | GGTTTAAUTTAAGCAGTCTTTGGCTT |
| 78 | [At1g63690](http://aramemnon.botanik.uni-koeln.de/seq_view.ep?x=0&y=0&search=At1g63690) | GGCTTAAUATGGATTCGCTTCGATTT | GGTTTAAUTCATTTCTCAGAACACTG |
| 79 | [At5g13750](http://aramemnon.botanik.uni-koeln.de/seq_view.ep?orgm=0&search=At5g13750&cat=0&term=1) | GGCTTAAUATGGCGGAAGAGTATGCC | GGTTTAAUCTACTGTTGTGTTTCAGC |
| 80 | [At4g00350](http://aramemnon.botanik.uni-koeln.de/seq_view.ep?orgm=0&search=At4g00350&cat=0&term=1) | GGCTTAAUATGGCTGACAAAGATGAA | GGTTTAAUTTAACGGTCGTTTTGGGC |
| 81 | [At5g13400](http://aramemnon.botanik.uni-koeln.de/seq_view.ep?orgm=0&search=At5g13400&cat=0&term=1) | GGCTTAAUATGGTTGCTTCTGAGATT | GGTTTAAUTTAAAGAACAGCACTACT |
| 82 | [At5g09220](http://aramemnon.botanik.uni-koeln.de/seq_view.ep?orgm=0&search=At5g09220&cat=0&term=1) | GGCTTAAUATGGGTGAAACCGCTGCC | GGTTTAAUTCAATATGTAGACTTGAA |
| 83 | [At1g57990](http://aramemnon.botanik.uni-koeln.de/seq_view.ep?orgm=0&search=At1g57990&cat=0&term=1) | GGCTTAAUATGGAGATGACCGAAGCT | GGTTTAAUCTAAACGTCAATATTGTT |
| 84 | [At1g12480](http://aramemnon.botanik.uni-koeln.de/seq_view.ep?orgm=0&search=At1g12480&cat=0&term=1) | GGCTTAAUATGGAGAGGAAACAGTCA | GGTTTAAUTCAGTGATGCGACTCTTC |
| 85 | [At2g03520](http://aramemnon.botanik.uni-koeln.de/seq_view.ep?orgm=0&search=At2g03520&cat=0&term=1) | GGCTTAAUAACTTATGTTTCTATGGT | GGTTTAAUGTACTCCATTGATCGTTT |
| 86 | [At4g22840](http://aramemnon.botanik.uni-koeln.de/seq_view.ep?orgm=0&search=At4g22840&cat=0&term=1) | GGCTTAAUATGAGCGTGATCACAACT | GGTTTAAUTTAAAATGTGTTACTCTT |
| 87 | [At1g12640](http://aramemnon.botanik.uni-koeln.de/seq_view.ep?orgm=0&search=At1g12640&cat=0&term=1) | GGCTTAAUATGGATATGAGTTCAATG | GGTTTAAUTTATTCTTCTTTACGCGG |
| 88 | [At1g33080](http://aramemnon.botanik.uni-koeln.de/seq_view.ep?orgm=0&search=At1g33080&cat=0&term=1) | GGCTTAAUATGGCGAGAAGAGAAGGA | GGTTTAAUTCATTCGTTTTGTAAAGT |
| 89 | [At4g13345](http://aramemnon.botanik.uni-koeln.de/seq_view.ep?orgm=0&search=At4g13345&cat=0&term=1) | GGCTTAAUATGGAAACCGGTACAAGC | GGTTTAAUTCATGTTGTTTGTCTACT |
| 90 | [At5g64290](http://aramemnon.botanik.uni-koeln.de/seq_view.ep?orgm=0&search=At5g64290&cat=0&term=1) | GGCTTAAUATGGAGAGTTTCGCTCTT | GGTTTAAUTTAGTAGAGACCCAAAAA |
| 91 | [At3g25410](http://aramemnon.botanik.uni-koeln.de/seq_view.ep?orgm=0&search=At3g25410&cat=0&term=1) | GGCTTAAUATGACTTTAATCGCTTCT | GGTTTAAUTCATGATTCAGCGGTGTA |
| 92 | [At3g25410](http://aramemnon.botanik.uni-koeln.de/seq_view.ep?orgm=0&search=At3g25410&cat=0&term=1) | GGCTTAAUATGACTTTAATCGCTTCT | GGTTTAAUTCATGATTCAGCGGTGTA |
| 93 | [At5g14850](http://aramemnon.botanik.uni-koeln.de/seq_view.ep?orgm=0&search=At5g14850&cat=0&term=1) | GGCTTAAUATGGATATAAGGAAGCGG | GGTTTAAUTTATGGGAATGCGTGGTT |
| 94 | [At1g63010](http://aramemnon.botanik.uni-koeln.de/seq_view.ep?orgm=0&search=At1g63010&cat=0&term=1) | GGCTTAAUTATTTTCATCGGTTTATT | GGTTTAAUTCAATAGAGTGAGTTATA |
| 95 | [At1g79820](http://aramemnon.botanik.uni-koeln.de/seq_view.ep?orgm=0&search=At1g79820&cat=0&term=1) | GGCTTAAUATGATTGTTGGAGCTTCT | GGTTTAAUTCACTGGGTGGAAGAGAG |
| 96 | [At5g55960](http://aramemnon.botanik.uni-koeln.de/seq_view.ep?orgm=0&search=At5g55960&cat=0&term=1) | GGCTTAAUATGGAATTGGTCCCGTAC | GGTTTAAUCTAATTGATCTTCTTTGG |
| 97 | [At5g26820](http://aramemnon.botanik.uni-koeln.de/seq_view.ep?orgm=0&search=At5g26820&cat=0&term=1) | GGCTTAAUATGGTTGTTTCAATGGCT | GGTTTAAUTCAATTTGAGAGAGGGTC |
| 98 | [At2g21340](http://aramemnon.botanik.uni-koeln.de/seq_view.ep?orgm=0&search=At2g21340&cat=0&term=1) | GGCTTAAUATGCAAATTCAATGCAAA | GGTTTAAUCTACGCAGCTTTCACTTT |
| 99 | [At5g13760](http://aramemnon.botanik.uni-koeln.de/seq_view.ep?orgm=0&search=At5g13760&cat=0&term=1) | GGCTTAAUATGGGTGCAACAGAGCCC | GGTTTAAUCTAAGCTAGAGCAAGGCG |
| 100 | [At5g13740](http://aramemnon.botanik.uni-koeln.de/seq_view.ep?orgm=0&search=At5g13740&cat=0&term=1) | GGCTTAAUATGAAGCAGCTCCGGCGA | GGTTTAAUTCATCTTCGACTCGTCGT |
| 101 | [At4g27720](http://aramemnon.botanik.uni-koeln.de/seq_view.ep?orgm=0&search=At4g27720&cat=0&term=1) | GGCTTAAUATGGAGATTTTCTACTAC | GGTTTAAUTCATATGTTGAGGGGATC |
| 102 | [At1g72130](http://aramemnon.botanik.uni-koeln.de/seq_view.ep?orgm=0&search=At1g72130&cat=0&term=1) | GGCTTAAUATGGCTATCACCTACTCC | GGTTTAAUTTAAAAGGTGTTTGATCT |
| 103 | [At5g03555](http://aramemnon.botanik.uni-koeln.de/seq_view.ep?orgm=0&search=At5g03555&cat=0&term=1) | GGCTTAAUATGACCGGCTCAGAAATT | GGTTTAAUTTACAAAAGCGGATGTGA |
| 104 | [At3g56200](http://aramemnon.botanik.uni-koeln.de/seq_view.ep?orgm=0&search=At3g56200&cat=0&term=1) | GGCTTAAUATGACACCACAGATCAAA | GGTTTAAUCTAGTTTGCTGCGAGGCT |
| 105 | [At4g27970](http://aramemnon.botanik.uni-koeln.de/seq_view.ep?orgm=0&search=At4g27970&cat=0&term=1) | GGCTTAAUATGAATAATCCAAGATCG | GGTTTAAUTTAATTTGAATTCTGAAC |
| 106 | [At5g38460](http://aramemnon.botanik.uni-koeln.de/seq_view.ep?orgm=0&search=At5g38460&cat=0&term=1) | GGCTTAAUATGCCGAAGAAGAAGCCG | GGTTTAAUTCAGATTTGCTTCTTTTC |
| 107 | [At5g10190](http://aramemnon.botanik.uni-koeln.de/seq_view.ep?orgm=0&search=At5g10190&cat=0&term=1) | GGCTTAAUATGAAGTCGGAGACTTTA | GGTTTAAUTTAACTCTCATTCTGATG |
| 108 | [At2g36630](http://aramemnon.botanik.uni-koeln.de/seq_view.ep?orgm=0&search=At2g36630&cat=0&term=1) | GGCTTAAUATGGGATTGTGGAATGGA | GGTTTAAUTCATTGACTGCTGCAGAA |
| 109 | [At4g36790](http://aramemnon.botanik.uni-koeln.de/seq_view.ep?orgm=0&search=At4g36790&cat=0&term=1) | GGCTTAAUATGGGTCATGCTCGAACT | GGTTTAAUTCAGATCATTTCAGTTTC |
| 110 | [At2g01170](http://aramemnon.botanik.uni-koeln.de/seq_view.ep?orgm=0&search=At2g01170&cat=0&term=1) | GGCTTAAUATGGGATTGGGCGGCGAT | GGTTTAAUTCAGCTAAGAATGTTGGA |
| 111 | [At5g46340](http://aramemnon.botanik.uni-koeln.de/seq_view.ep?orgm=0&search=At5g46340&cat=0&term=1) | GGCTTAAUATGGTGGATCCTGGACCA | GGTTTAAUCTAACGATGAATCTGAAG |
| 112 | [At5g49990](http://aramemnon.botanik.uni-koeln.de/seq_view.ep?orgm=0&search=At5g49990&cat=0&term=1) | GGCTTAAUATGTCAGCTCCGAAATCG | GGTTTAAUTCAGACAGAAGGGAAATA |
| 113 | [At3g10960](http://aramemnon.botanik.uni-koeln.de/seq_view.ep?orgm=0&search=At3g10960&cat=0&term=1) | GGCTTAAUATGGAGCAACAGCAACAA | GGTTTAAUCTAAACGGTAGTATCAAT |
| 114 | [At2g04100](http://aramemnon.botanik.uni-koeln.de/seq_view.ep?orgm=0&search=At2g04100&cat=0&term=1) | GGCTTAAUATGGAAGATCCACTTTTA | GGTTTAAUTCAAGCAAGTCCATTGCC |
| 115 | [At3g59310](http://aramemnon.botanik.uni-koeln.de/seq_view.ep?orgm=0&search=At3g59310&cat=0&term=1) | GGCTTAAUATGGGTTTCGATTTAAAG | GGTTTAAUTTAGGTTCCCGGCTCATC |
| 116 | [At3g60070](http://aramemnon.botanik.uni-koeln.de/seq_view.ep?orgm=0&search=At3g60070&cat=0&term=1) | GGCTTAAUATGACGTCATCGGTTATT | GGTTTAAUTCATTCTAGTAGTAGCGG |
| 117 | [At4g22990](http://aramemnon.botanik.uni-koeln.de/seq_view.ep?orgm=0&search=At4g22990&cat=0&term=1) | GGCTTAAUATGGTAGCCTTCGGGAAA | GGTTTAAUCTAATACAAGGAGTTATA |
| 118 | [At1g68570](http://aramemnon.botanik.uni-koeln.de/seq_view.ep?orgm=0&search=At1g68570&cat=0&term=1) | GGCTTAAUATGGAGGAGCAAAGCAAG | GGTTTAAUTCATTCATCAACTAAACT |
| 119 | [At5g52540](http://aramemnon.botanik.uni-koeln.de/seq_view.ep?orgm=0&search=At5g52540&cat=0&term=1) | GGCTTAAUATGGCGACGAGCTTGCCA | GGTTTAAUTCACATGAACTTAAGAAC |
| 120 | [At5g51710](http://aramemnon.botanik.uni-koeln.de/seq_view.ep?orgm=0&search=At5g51710&cat=0&term=1) | GGCTTAAUATGGCGAGATTCGCAGTG | GGTTTAAUTCACTTGGTTCTGTTATG |
| 121 | [At2g01420](http://aramemnon.botanik.uni-koeln.de/seq_view.ep?orgm=0&search=At2g01420&cat=0&term=1) | GGCTTAAUATGATTACGTGGCACGAC | GGTTTAAUTCAAAGGCCAAGAAGAAT |
| 122 | [At5g63850](http://aramemnon.botanik.uni-koeln.de/seq_view.ep?orgm=0&search=At5g63850&cat=0&term=1) | GGCTTAAUATGGATGTTCCACGGCCA | GGTTTAAUTTAGTAAGTAGTCTTGAA |
| 123 | [At4g04340](http://aramemnon.botanik.uni-koeln.de/seq_view.ep?orgm=0&search=At4g04340&cat=0&term=1) | GGCTTAAUATGGCAACACTTAAAGAC | GGTTTAAUCTAGACTTCTTTACCGTT |
| 124 | [At5g02410](http://aramemnon.botanik.uni-koeln.de/seq_view.ep?orgm=0&search=At5g02410&cat=0&term=1) | GGCTTAAUATGGGGAAATTAGCCGTT | GGTTTAAUCTACCATATAAACCTCTG |
| 125 | [At2g27810](http://aramemnon.botanik.uni-koeln.de/seq_view.ep?orgm=0&search=At2g27810&cat=0&term=1) | GGCTTAAUATGTCAAGCTCCGACCCG | GGTTTAAUTCAAATGCCCACCCATTT |
| 126 | [At4g35300](http://aramemnon.botanik.uni-koeln.de/seq_view.ep?orgm=0&search=At4g35300&cat=0&term=1) | GGCTTAAUATGAGTGGAGCTGTGCTT | GGTTTAAUTCACTCGTTTTTAGCAGC |
| 127 | [At1g18010](http://aramemnon.botanik.uni-koeln.de/seq_view.ep?orgm=0&search=At1g18010&cat=0&term=1) | GGCTTAAUATGAATGTGAGAGACGAG | GGTTTAAUCTAAACCTTATCATTACT |
| 128 | [At1g50430](http://aramemnon.botanik.uni-koeln.de/seq_view.ep?orgm=0&search=At1g50430&cat=0&term=1) | GGCTTAAUATGGCGGAGACTGTACAT | GGTTTAAUTCAATAAATTCCCGGAAT |
| 129 | [At3g16180](http://aramemnon.botanik.uni-koeln.de/seq_view.ep?orgm=0&search=At3g16180&cat=0&term=1) | GGCTTAAUATGGAGAACCCTCCGGAT | GGTTTAAUTTAGTTTAATTTAATAACTTCCTCTTCTTCTTTTCTCATACCATTAACCTTATCATTC |
| 130 | [At1g15500](http://aramemnon.botanik.uni-koeln.de/seq_view.ep?orgm=0&search=At1g15500&cat=0&term=1) | GGCTTAAUATGGAAGGTCTGATTCAA | GGTTTAAUCTAAATGCCAGTAGGAGT |
| 131 | [At5g17700](http://aramemnon.botanik.uni-koeln.de/seq_view.ep?orgm=0&search=At5g17700&cat=0&term=1) | GGCTTAAUATGAGTGGAGGTGGTGGA | GGTTTAAUTCACTTTCTCTCTTCATC |
| 132 | [At3g02690](http://aramemnon.botanik.uni-koeln.de/seq_view.ep?orgm=0&search=At3g02690&cat=0&term=1) | GGCTTAAUATGGAGTGGCCATGGTCG | GGTTTAAUTCAGTCGTTGCCTTCGGG |
| 133 | [At5g57090](http://aramemnon.botanik.uni-koeln.de/seq_view.ep?orgm=0&search=At5g57090&cat=0&term=1) | GGCTTAAUATGATCACCGGCAAAGAC | GGTTTAAUTTAAAGCCCCAAAAGAAC |
| 134 | [At1g30220](http://aramemnon.botanik.uni-koeln.de/seq_view.ep?orgm=0&search=At1g30220&cat=0&term=1) | GGCTTAAUATGGAGGGAGGAATAATA | GGTTTAAUTCATGCACTCTGGTTTTG |
| 135 | [At3g21690](http://aramemnon.botanik.uni-koeln.de/seq_view.ep?orgm=0&search=At3g21690&cat=0&term=1) | GGCTTAAUATGGACTCGTCTCCAAAC | GGTTTAAUTCATTCAGGAACAACTTC |
| 136 | [At1g76520](http://aramemnon.botanik.uni-koeln.de/seq_view.ep?orgm=0&search=At1g76520&cat=0&term=1) | GGCTTAAUATGGTGAAGCTTTTGGAG | GGTTTAAUCTAAGCTACAAGCCACAT |
| 137 | [At1g77380](http://aramemnon.botanik.uni-koeln.de/seq_view.ep?orgm=0&search=At1g77380&cat=0&term=1) | GGCTTAAUATGGTTCAAAACCACCAA | GGTTTAAUTCAGTATTCGCTTCGAAA |
| 138 | [At2g26690](http://aramemnon.botanik.uni-koeln.de/seq_view.ep?orgm=0&search=At2g26690&cat=0&term=1) | GGCTTAAUATGGAGAGCAAAGGGAGT | GGTTTAAUTCAGCAGTCTTCAACTGA |
| 139 | [At5g65000](http://aramemnon.botanik.uni-koeln.de/seq_view.ep?orgm=0&search=At5g65000&cat=0&term=1) | GGCTTAAUATGGCGACGGCTAACGGA | GGTTTAAUTTACACCTTCTTCTTCTT |
| 140 | [At1g57600](http://aramemnon.botanik.uni-koeln.de/seq_view.ep?orgm=0&search=At1g57600&cat=0&term=1) | GGCTTAAUATGAACACTCACAATAAT | GGTTTAAUCTACTTCGGGCTATGCGC |
| 141 | [At1g55620](http://aramemnon.botanik.uni-koeln.de/seq_view.ep?orgm=0&search=At1g55620&cat=0&term=1) | GGCTTAAUATGATGCACGGTTTGCTT | GGTTTAAUTCAATGCCCATTTGTACC |
| 142 | [At1g63050](http://aramemnon.botanik.uni-koeln.de/seq_view.ep?orgm=0&search=At1g63050&cat=0&term=1) | GGCTTAAUATGGAATTGCTTGACATG | GGTTTAAUTTATTCTTCTTTTCTGGT |
| 143 | [At5g64410](http://aramemnon.botanik.uni-koeln.de/seq_view.ep?orgm=0&search=At5g64410&cat=0&term=1) | GGCTTAAUATGGCCACCGCCGACGAA | GGTTTAAUTTATTTAACCGGACAACC |
| 144 | [At3g21620](http://aramemnon.botanik.uni-koeln.de/seq_view.ep?orgm=0&search=At3g21620&cat=0&term=1) | GGCTTAAUATGGACGAGGTGATGACA | GGTTTAAUCTAGCTTGAGCTCCTCAT |
| 145 | [At5g04770](http://aramemnon.botanik.uni-koeln.de/seq_view.ep?orgm=0&search=At5g04770&cat=0&term=1) | GGCTTAAUATGGAGGTCCAAAGCAGC | GGTTTAAUTCACACTTCAATTAGCTC |
| 146 | [At3g59360](http://aramemnon.botanik.uni-koeln.de/seq_view.ep?orgm=0&search=At3g59360&cat=0&term=1) | GGCTTAAUATGAAGAACGGTATAGCT | GGTTTAAUCTATCTGGGAAGCAGTGG |
| 147 | [At1g59740](http://aramemnon.botanik.uni-koeln.de/seq_view.ep?orgm=0&search=At1g59740&cat=0&term=1) | GGCTTAAUATGGCAGAGATAAACAAA | GGTTTAAUCTAAATGTTCTCATCACC |
| 148 | [At4g26590](http://aramemnon.botanik.uni-koeln.de/seq_view.ep?orgm=0&search=At4g26590&cat=0&term=1) | GGCTTAAUATGGTAGGCTCTCTCGAA | GGTTTAAUTTAGAACACCGGGCAGCC |
| 149 | [At3g55640](http://aramemnon.botanik.uni-koeln.de/seq_view.ep?orgm=0&search=At3g55640&cat=0&term=1) | GGCTTAAUATGGTGATGCAGACGGAA | GGTTTAAUTTATAGATTCGAAGAAAG |
| 150 | [At1g15180](http://aramemnon.botanik.uni-koeln.de/seq_view.ep?orgm=0&search=At1g15180&cat=0&term=1) | GGCTTAAUATGGGAGACGCAGAGAGC | GGTTTAAUTTACGTTCCATAGGCCAA |
| 151 | [At5g52450](http://aramemnon.botanik.uni-koeln.de/seq_view.ep?orgm=0&search=At5g52450&cat=0&term=1) | GGCTTAAUATGAGGGACGACAGAGAA | GGTTTAAUTCAAAATACAACAACTGA |
| 152 | [At5g46110](http://aramemnon.botanik.uni-koeln.de/seq_view.ep?orgm=0&search=At5g46110&cat=0&term=1) | GGCTTAAUATGGAGTCACGCGTGCTG | GGTTTAAUCTATGCTTTCTTTCCTTG |
| 153 | [At1g10540](http://aramemnon.botanik.uni-koeln.de/seq_view.ep?orgm=0&search=At1g10540&cat=0&term=1) | GGCTTAAUATGGCAGGTGATGGCGTA | GGTTTAAUTCAGTGAGAAGGAAAGTA |
| 154 | [At1g27040](http://aramemnon.botanik.uni-koeln.de/seq_view.ep?orgm=0&search=At1g27040&cat=0&term=1) | GGCTTAAUATGGAAGTAGAAATGCAT | GGTTTAAUTCAACTTATTGAACCAGT |
| 155 | [At3g05400](http://aramemnon.botanik.uni-koeln.de/seq_view.ep?orgm=0&search=At3g05400&cat=0&term=1) | GGCTTAAUATGGAAGGAGAGAATAAT | GGTTTAAUCTAAACATTGACAAACGA |
| 156 | [At4g32400](http://aramemnon.botanik.uni-koeln.de/seq_view.ep?orgm=0&search=At4g32400&cat=0&term=1) | GGCTTAAUATGGGGAAAACCGGAATC | GGTTTAAUTCAAGCTTCTTGGTTGTT |
| 157 | [At1g73590](http://aramemnon.botanik.uni-koeln.de/seq_view.ep?orgm=0&search=At1g73590&cat=0&term=1) | GGCTTAAUATGATTACGGCGGCGGAC | GGTTTAAUTCATAGACCCAAGAGAAT |
| 158 | [At5g62890](http://aramemnon.botanik.uni-koeln.de/seq_view.ep?orgm=0&search=At1g73590&cat=0&term=1) | GGCTTAAUTTGGGTCTCGTTGGTTTT | GGTTTAAUTTAGACAGATGGGAAGTA |
| 159 | [At1g78560](http://aramemnon.botanik.uni-koeln.de/seq_view.ep?orgm=0&search=At1g78560&cat=0&term=1) | GGCTTAAUATGGCGTCGGCGATTTCT | GGTTTAAUTTAGTCTTCAAGCTGTTT |
| 160 | [At1g44100](http://aramemnon.botanik.uni-koeln.de/seq_view.ep?orgm=0&search=At1g44100&cat=0&term=1) | GGCTTAAUATGGTCGTTCAGAATGTT | GGTTTAAUTCAGAATTCAGACTGGAA |
| 161 | [At4g24120](http://aramemnon.botanik.uni-koeln.de/seq_view.ep?orgm=0&search=At4g24120&cat=0&term=1) | GGCTTAAUATGAAGAGAGAAGGAGAA | GGTTTAAUCTATGAAGCTAAGAACTT |
| 162 | [At1g12110](http://aramemnon.botanik.uni-koeln.de/seq_view.ep?orgm=0&search=At1g12110&cat=0&term=1) | GGCTTAAUATGTCTCTTCCTGAAACT | GGTTTAAUTCAATGACCCATTGGAAT |
| 163 | [At3g23560](http://aramemnon.botanik.uni-koeln.de/seq_view.ep?orgm=0&search=At3g23560&cat=0&term=1) | GGCTTAAUATGTCAGGTCATCAGTGC | GGTTTAAUTCACTTGAGATAGTCGAT |
| 164 | [At2g48020](http://aramemnon.botanik.uni-koeln.de/seq_view.ep?orgm=0&search=At2g48020&cat=0&term=1) | GGCTTAAUATGTCCAAGGCAAGTGAT | GGTTTAAUCTATGGATTGACTATAGC |
| 165 | [At3g27020](http://aramemnon.botanik.uni-koeln.de/seq_view.ep?orgm=0&search=At3g27020&cat=0&term=1) | GGCTTAAUATGGGGACGGAGATCCCT | GGTTTAAUCTATCTTGCTGAGGACGG |
| 166 | [At1g48640](http://aramemnon.botanik.uni-koeln.de/seq_view.ep?orgm=0&search=At1g48640&cat=0&term=1) | GGCTTAAUATGGATTTCGTTAGGGGT | GGTTTAAUCTATTGCGGCATATTAGC |
| 167 | [At5g01500](http://aramemnon.botanik.uni-koeln.de/seq_view.ep?orgm=0&search=At5g01500&cat=0&term=1) | GGCTTAAUATGGGAGAAGAGAAGTCT | GGTTTAAUTCAGGTTTGTTCATCGAT |
| 168 | [At3g18830](http://aramemnon.botanik.uni-koeln.de/seq_view.ep?orgm=0&search=At3g18830&cat=0&term=1) | GGCTTAAUATGATCGCCGCAATCGGA | GGTTTAAUCTACGAACTTTGTGTGTC |
| 169 | [At2g41700](http://aramemnon.botanik.uni-koeln.de/seq_view.ep?orgm=0&search=At2g41700&cat=0&term=1) | GGCTTAAUATGATTGGAGATCCTCCT | GGTTTAAUCTATGAGTTAGCTGCAAA |
| 170 | [At1g73220](http://aramemnon.botanik.uni-koeln.de/seq_view.ep?orgm=0&search=At1g73220&cat=0&term=1) | GGCTTAAUATGGAACCTTCAAAACAA | GGTTTAAUTCAAGTAATCATGATTGT |
| 171 | [At1g14560](http://aramemnon.botanik.uni-koeln.de/seq_view.ep?orgm=0&search=At1g14560&cat=0&term=1) | GGCTTAAUATGGCTTATAAAGAAGGG | GGTTTAAUTCATGCTGGCTTTGATCT |
| 172 | [At3g45040](http://aramemnon.botanik.uni-koeln.de/seq_view.ep?orgm=0&search=At3g45040&cat=0&term=1) | GGCTTAAUATGAAGACGACGGCGACG | GGTTTAAUTTACAAGCAGAGGAGTGA |
| 173 | [At4g25640](http://aramemnon.botanik.uni-koeln.de/seq_view.ep?orgm=0&search=At4g25640&cat=0&term=1) | GGCTTAAUATGGATCCGACGGCGCCG | GGTTTAAUTCACGCAAGTATATCCTT |
| 174 | [At3g01550](http://aramemnon.botanik.uni-koeln.de/seq_view.ep?orgm=0&search=At3g01550&cat=0&term=1) | GGCTTAAUATGTTCGCTCTCACATTT | GGTTTAAUTCAAGACATTTTTGGATT |
| 175 | [At1g70940](http://aramemnon.botanik.uni-koeln.de/seq_view.ep?orgm=0&search=At1g70940&cat=0&term=1) | GGCTTAAUATGATCTCATGGCACGAC | GGTTTAAUTTATAACCCGAGTAGAAT |
| 176 | [At3g53940](http://aramemnon.botanik.uni-koeln.de/seq_view.ep?orgm=0&search=At3g53940&cat=0&term=1) | GGCTTAAUATGGAAGCTCGAGTCGGT | GGTTTAAUTTAATTAGGGACAGTTGA |
| 177 | [At1g54730](http://aramemnon.botanik.uni-koeln.de/seq_view.ep?orgm=0&search=At1g54730&cat=0&term=1) | GGCTTAAUATGATCGGCGCAGCTATG | GGTTTAAUTTAAGACATCGATCGGAT |
| 178 | [At1g79410](http://aramemnon.botanik.uni-koeln.de/seq_view.ep?orgm=0&search=At1g79410&cat=0&term=1) | GGCTTAAUATGATCCCTGACGGTTCC | GGTTTAAUTCAGCAACTATGGCTAGT |
| 179 | [At4g38640](http://aramemnon.botanik.uni-koeln.de/seq_view.ep?orgm=0&search=At4g38640&cat=0&term=1) | GGCTTAAUATGGCGGCGGACGACGGC | GGTTTAAUCTATGCATTAAGAGCATT |
| 180 | [At1g33110](http://aramemnon.botanik.uni-koeln.de/seq_view.ep?orgm=0&search=At1g33110&cat=0&term=1) | GGCTTAAUATGGCCGGAGGAGGAGGA | GGTTTAAUTTATTCCTCTGATGATAC |
| 181 | [At4g31600](http://aramemnon.botanik.uni-koeln.de/seq_view.ep?orgm=0&search=At4g31600&cat=0&term=1) | GGCTTAAUATGGAGGTTCAAGCTGAA | GGTTTAAUTCATTTTTTGTGAGCTTC |
| 182 | [At1g15170](http://aramemnon.botanik.uni-koeln.de/seq_view.ep?orgm=0&search=At1g15170&cat=0&term=1) | GGCTTAAUATGGGAGACGCAGAGAGC | GGTTTAAUTTATGTTCCATAGGCCAA |
| 183 | [At5g24380](http://aramemnon.botanik.uni-koeln.de/seq_view.ep?orgm=0&search=At5g24380&cat=0&term=1) | GGCTTAAUATGACATATGTTGGAGCT | GGTTTAAUTTAATGAGCCGCAGTGAA |
| 184 | [At3g08040](http://aramemnon.botanik.uni-koeln.de/seq_view.ep?orgm=0&search=At3g08040&cat=0&term=1) | GGCTTAAUATGACGGAAACTGGTGAT | GGTTTAAUCTAGGAAGATGAAGAGGA |
| 185 | [At4g32140](http://aramemnon.botanik.uni-koeln.de/seq_view.ep?orgm=0&search=At4g32140&cat=0&term=1) | GGCTTAAUATGGGTTGGAGATACAAA | GGTTTAAUCTACAATCCAAGCTTCTT |
| 186 | [At2g34960](http://aramemnon.botanik.uni-koeln.de/seq_view.ep?orgm=0&search=At2g34960&cat=0&term=1) | GGCTTAAUATGCTCTCTGTCTTCTGC | GGTTTAAUTTATGTTCTTGGCACAAT |
| 187 | [At1g08930](http://aramemnon.botanik.uni-koeln.de/seq_view.ep?orgm=0&search=At1g08930&cat=0&term=1) | GGCTTAAUATGGAGAGACAAAAGAGC | GGTTTAAUTTATTGCACAGAGTTGTT |
| 188 | [At5g41800](http://aramemnon.botanik.uni-koeln.de/seq_view.ep?orgm=0&search=At5g41800&cat=0&term=1) | GGCTTAAUATGACGGATCCTCCCAGA | GGTTTAAUCTAATCAACAACTTCACT |
| 189 | [At1g11670](http://aramemnon.botanik.uni-koeln.de/seq_view.ep?orgm=0&search=At1g11670&cat=0&term=1) | GGCTTAAUATGGGTTCGGAAGCGACC | GGTTTAAUTTATTGCTTAAGAAGCGG |
| 190 | [At5g61520](http://aramemnon.botanik.uni-koeln.de/seq_view.ep?orgm=0&search=At5g61520&cat=0&term=1) | GGCTTAAUATGGCGCCGGCAAAATAC | GGTTTAAUTCAATGGCTAAGAATGGT |
| 191 | [At3g21390](http://aramemnon.botanik.uni-koeln.de/seq_view.ep?orgm=0&search=At3g21390&cat=0&term=1) | GGCTTAAUATGAGTGGTACAGAGTTA | GGTTTAAUTCAGGTCAAATTTGCCTC |
| 192 | [At3g03090](http://aramemnon.botanik.uni-koeln.de/seq_view.ep?orgm=0&search=At3g03090&cat=0&term=1) | GGCTTAAUATGGGGTTTGATCCCGAG | GGTTTAAUTTAGAGACATTTGGCTTC |
| 193 | [At3g59340](http://aramemnon.botanik.uni-koeln.de/seq_view.ep?orgm=0&search=At3g59340&cat=0&term=1) | GGCTTAAUATGGCAATGGGTTTCGAT | GGTTTAAUCTATAACGATGTGTCTGG |
| 194 | [At5g12860](http://aramemnon.botanik.uni-koeln.de/seq_view.ep?orgm=0&search=At5g12860&cat=0&term=1) | GGCTTAAUATGGGATTAGGAGCTTCC | GGTTTAAUTCACCACAAGCCAATGAA |
| 195 | [At4g16370](http://aramemnon.botanik.uni-koeln.de/seq_view.ep?orgm=0&search=At4g16370&cat=0&term=1) | GGCTTAAUATGGACGCGGAGAAGGCT | GGTTTAAUTTAGAAAACGGGACAGCC |
| 196 | [At3g55740](http://aramemnon.botanik.uni-koeln.de/seq_view.ep?orgm=0&search=At3g55740&cat=0&term=1) | GGCTTAAUATGGATACGAGTGAAGCA | GGTTTAAUTCAAACATCAGCAAAAAC |
| 197 | [At1g31830](http://aramemnon.botanik.uni-koeln.de/seq_view.ep?orgm=0&search=At1g31830&cat=0&term=1) | GGCTTAAUATGCAGAAGCGGAGAATC | GGTTTAAUTTAACGTATTAGAGTTTC |
| 198 | [At4g02050](http://aramemnon.botanik.uni-koeln.de/seq_view.ep?orgm=0&search=At4g02050&cat=0&term=1) | GGCTTAAUATGGCAGGAGGGTCGTTT | GGTTTAAUTTAAACAGATACATTCTT |
| 199 | [At5g44370](http://aramemnon.botanik.uni-koeln.de/seq_view.ep?orgm=0&search=At5g44370&cat=0&term=1) | GGCTTAAUATGAAGTTATCAAATATT | GGTTTAAUTCAATCAAAGATCCTTTC |
| 200 | [At2g02020](http://aramemnon.botanik.uni-koeln.de/seq_view.ep?orgm=0&search=At2g02020&cat=0&term=1) | GGCTTAAUATGGCTTCCATTGATGAA | GGTTTAAUTCAATTCAAACAAGAAAT |
| 201 | [At4g22790](http://aramemnon.botanik.uni-koeln.de/seq_view.ep?orgm=0&search=At4g22790&cat=0&term=1) | GGCTTAAUATGTCAGAAACATCAAAG | GGTTTAAUCTATGAGTGGCTATCTTG |
| 202 | [At1g65730](http://aramemnon.botanik.uni-koeln.de/seq_view.ep?orgm=0&search=At1g65730&cat=0&term=1) | GGCTTAAUATGGAAGTCGAACGATCG | GGTTTAAUTTAAGAAGGGTTCAAGAA |
| 203 | [At5g01180](http://aramemnon.botanik.uni-koeln.de/seq_view.ep?orgm=0&search=At5g01180&cat=0&term=1) | GGCTTAAUAAACAAATGAATATGGAA | GGTTTAAUTCAAAGCGCATGCCCGGT |
| 204 | [At5g01990](http://aramemnon.botanik.uni-koeln.de/seq_view.ep?orgm=0&search=At5g01990&cat=0&term=1) | GGCTTAAUATGATTGCTCGGATCCTT | GGTTTAAUTCAGAAGAGTATGTTAAT |
| 205 | [At3g54510](http://aramemnon.botanik.uni-koeln.de/seq_view.ep?orgm=0&search=At3g54510&cat=0&term=1) | GGCTTAAUATGTGCAGAATTAGATTC | GGTTTAAUTCAAATGGAGTCTGTCCC |
| 206 | [At1g62280](http://aramemnon.botanik.uni-koeln.de/seq_view.ep?orgm=0&search=At1g62280&cat=0&term=1) | GGCTTAAUATGGAAATTCCGAGGCAA | GGTTTAAUCTAGTTTTGGTTAGTCGC |
| 207 | [At4g35180](http://aramemnon.botanik.uni-koeln.de/seq_view.ep?orgm=0&search=At4g35180&cat=0&term=1) | GGCTTAAUATGTCACCAGCCCCCTCC | GGTTTAAUTTAGGGTCTGAAGAAGTT |
| 208 | [At2g36590](http://aramemnon.botanik.uni-koeln.de/seq_view.ep?orgm=0&search=At2g36590&cat=0&term=1) | GGCTTAAUATGAACTCTAAGAATCGC | GGTTTAAUTTACAAATCTGCAAAAAC |
| 209 | [At2g38330](http://aramemnon.botanik.uni-koeln.de/seq_view.ep?orgm=0&search=At2g38330&cat=0&term=1) | GGCTTAAUATGGCGGCGGTCGCTACC | GGTTTAAUTCATTCTGGCTTCTCTGG |
| 210 | [At1g30840](http://aramemnon.botanik.uni-koeln.de/seq_view.ep?orgm=0&search=At1g30840&cat=0&term=1) | GGCTTAAUATGAGTGATGGTCGAGTT | GGTTTAAUTCAAGCCCTATCATCCGC |
| 211 | [At5g41760](http://aramemnon.botanik.uni-koeln.de/seq_view.ep?orgm=0&search=At5g41760&cat=0&term=1) | GGCTTAAUATGGCGGCTACTCCGTGG | GGTTTAAUTCAAGAATCTGTCTTTTC |
| 212 | [At4g16480](http://aramemnon.botanik.uni-koeln.de/seq_view.ep?orgm=0&search=At4g16480&cat=0&term=1) | GGCTTAAUATGGTGGAAGGAGGAATT | GGTTTAAUTTAAGCAGCATCGACTTC |
| 213 | [At2g28780](http://aramemnon.botanik.uni-koeln.de/seq_view.ep?orgm=0&search=At2g28780&cat=0&term=1) | GGCTTAAUATGCTAATGACGGAGAGA | GGTTTAAUTCATTTATACAAAGAACG |
| 214 | [At3g45700](http://aramemnon.botanik.uni-koeln.de/seq_view.ep?orgm=0&search=At3g45700&cat=0&term=1) | GGCTTAAUATGGCTAATTCAGACTCT | GGTTTAAUCTAGTTTGTAACATCTTT |
| 215 | [At1g12500](http://aramemnon.botanik.uni-koeln.de/seq_view.ep?orgm=0&search=At1g12500&cat=0&term=1) | GGCTTAAUATGGTTGAAGCTCAATCA | GGTTTAAUTCACTTCTGGTTTAGCAA |
| 216 | [At3g07080](http://aramemnon.botanik.uni-koeln.de/seq_view.ep?orgm=0&search=At3g07080&cat=0&term=1) protein | GGCTTAAUATGTCAACAAAGTTATGG | GGTTTAAUTTATACTACAGTCAGTGA |
| 217 | [At1g28230](http://aramemnon.botanik.uni-koeln.de/seq_view.ep?orgm=0&search=At1g28230&cat=0&term=1) | GGCTTAAUATGAAGAATGGTTTGATA | GGTTTAAUTTAAGCAACATAATCACT |
| 218 | [At5g36940](http://aramemnon.botanik.uni-koeln.de/seq_view.ep?orgm=0&search=At5g36940&cat=0&term=1) | GGCTTAAUATGGGTTGTTTAAGAAGC | GGTTTAAUTTAAGCCAAAGAATGTCC |
| 219 | [At2g43240](http://aramemnon.botanik.uni-koeln.de/seq_view.ep?orgm=0&search=At2g43240&cat=0&term=1) | GGCTTAAUATGAAGAACGGGATGGCT | GGTTTAAUTTATCTGGGAAGAAGTGG |
| 220 | [At5g52050](http://aramemnon.botanik.uni-koeln.de/seq_view.ep?orgm=0&search=At5g52050&cat=0&term=1) | GGCTTAAUATGAGTCAATCAAATCGT | GGTTTAAUCTACTTATCAACCATCCC |
| 221 | [At4g36670](http://aramemnon.botanik.uni-koeln.de/seq_view.ep?orgm=0&search=At4g36670&cat=0&term=1) | GGCTTAAUATGAACGCCACCGTGTCG | GGTTTAAUCTAAGCTGCACCGTTTTC |
| 222 | [At4g21680](http://aramemnon.botanik.uni-koeln.de/seq_view.ep?orgm=0&search=At4g21680&cat=0&term=1) | GGCTTAAUATGGATCAAAAAGTTAGA | GGTTTAAUTCAGACTTCCTCCTCTTC |
| 223 | [At2g04080](http://aramemnon.botanik.uni-koeln.de/seq_view.ep?orgm=0&search=At2g04080&cat=0&term=1) | GGCTTAAUATGGAAGAGCCATTTCTT | GGTTTAAUTTAAACCAATCCATTTTC |
| 224 | [At4g04750](http://aramemnon.botanik.uni-koeln.de/seq_view.ep?orgm=0&search=At4g04750&cat=0&term=1) | GGCTTAAUATGCAGTTATTTGTCGGT | GGTTTAAUCTAATGAGAGCCATTATT |
| 225 | [At4g34100](http://aramemnon.botanik.uni-koeln.de/seq_view.ep?orgm=0&search=At4g34100&cat=0&term=1) | GGCTTAAUATGAAACTCCTAACGGCT | GGTTTAAUCTAAGCTTCTTGTTGGAT |
| 226 | [At1g34580](http://aramemnon.botanik.uni-koeln.de/seq_view.ep?orgm=0&search=At1g34580&cat=0&term=1) | GGCTTAAUATGGCCGGTGGAGGATTG | GGTTTAAUTTAAGTTGACGTCGGTTT |
| 227 | [At4g38050](http://aramemnon.botanik.uni-koeln.de/seq_view.ep?orgm=0&search=At4g38050&cat=0&term=1) | GGCTTAAUATGAGAGAACTACAGGGG | GGTTTAAUTTAACAACATCTGCAACC |
| 228 | [At1g57980](http://aramemnon.botanik.uni-koeln.de/seq_view.ep?orgm=0&search=At1g57980&cat=0&term=1) | GGCTTAAUATGGAGATGACCGAAGCT | GGTTTAAUCTAAACTTCTACATTGTT |
| 229 | [At1g77210](http://aramemnon.botanik.uni-koeln.de/seq_view.ep?orgm=0&search=At1g77210&cat=0&term=1) | GGCTTAAUATGGCCGGTGGAGCTCTT | GGTTTAAUTTATTCATCAACATCTTC |
| 230 | [At5g05630](http://aramemnon.botanik.uni-koeln.de/seq_view.ep?orgm=0&search=At5g05630&cat=0&term=1) | GGCTTAAUATGACTGAGCTTAGCTCT | GGTTTAAUTTACTCCATCAGGTTTGG |
| 231 | [At4g18210](http://aramemnon.botanik.uni-koeln.de/seq_view.ep?orgm=0&search=At4g18210&cat=0&term=1) | GGCTTAAUATGACGGCGGATCAAGAA | GGTTTAAUGGGTAGAAACGGGTAAGT |
| 232 | [At5g59740](http://aramemnon.botanik.uni-koeln.de/seq_view.ep?orgm=0&search=At5g59740&cat=0&term=1) | GGCTTAAUATGGCTGAGCCAGAATTA | GGTTTAAUTTAAGAGCTCTCAACCTT |
| 233 | [At1g72140](http://aramemnon.botanik.uni-koeln.de/seq_view.ep?orgm=0&search=At1g72140&cat=0&term=1) | GGCTTAAUATGTCGACATCCATCGGC | GGTTTAAUCTACTTTGGGCTGTTGTA |
| 234 | [At4g05120](http://aramemnon.botanik.uni-koeln.de/seq_view.ep?orgm=0&search=At4g05120&cat=0&term=1) | GGCTTAAUATGGCGGATAGATATGAG | GGTTTAAUTCAAAAGGCATTCTTCTT |
| 235 | [At5g23270](http://aramemnon.botanik.uni-koeln.de/seq_view.ep?orgm=0&search=At5g23270&cat=0&term=1) | GGCTTAAUATGGCAGGAGGGGCATTT | GGTTTAAUCTAAAAATAAGCATCATC |
| 236 | [At1g58340](http://aramemnon.botanik.uni-koeln.de/seq_view.ep?orgm=0&search=At1g58340&cat=0&term=1) | GGCTTAAUATGTGTAATTCAAAACCA | GGTTTAAUCTAAACCAACATGGTTCT |
| 237 | [At2g16990](http://aramemnon.botanik.uni-koeln.de/seq_view.ep?orgm=0&search=At2g16990&cat=0&term=1) | GGCTTAAUATGGGATTAAAACAATCC | GGTTTAAUCTAAACCAAGAAAGCAGA |
| 238 | [At3g20660](http://aramemnon.botanik.uni-koeln.de/seq_view.ep?orgm=0&search=At3g20660&cat=0&term=1) | GGCTTAAUATGGAATCTCCGGAGGAT | GGTTTAAUTTAACATATTACTTCTCC |
| 239 | [At1g66570](http://aramemnon.botanik.uni-koeln.de/seq_view.ep?orgm=0&search=At1g66570&cat=0&term=1) | GGCTTAAUATGAGTGACCTCCAAGCA | GGTTTAAUTTAAGGTAAAACGGTAAA |
